# Genome-Wide Analysis of Transcriptional Reprogramming in Mouse Models of Acute Myeloid Leukaemia

**DOI:** 10.1371/journal.pone.0016330

**Published:** 2011-01-28

**Authors:** Nicolas Bonadies, Samuel D. Foster, Wai-In Chan, Brynn T. Kvinlaug, Dominik Spensberger, Mark A. Dawson, Elaine Spooncer, Anthony D. Whetton, Andrew J. Bannister, Brian J. Huntly, Berthold Göttgens

**Affiliations:** 1 Department of Haematology, Cambridge Institute for Medical Research, Cambridge University, Cambridge, United Kingdom; 2 Gurdon Institute and Department of Pathology, Cambridge University, Cambridge, United Kingdom; 3 School of Cancer and Imaging Sciences, University of Manchester, Manchester, United Kingdom; Ludwig-Maximilians-Universität Müuchen, Germany

## Abstract

Acute leukaemias are commonly caused by mutations that corrupt the transcriptional circuitry of haematopoietic stem/progenitor cells. However, the mechanisms underlying large-scale transcriptional reprogramming remain largely unknown. Here we investigated transcriptional reprogramming at genome-scale in mouse retroviral transplant models of acute myeloid leukaemia (AML) using both gene-expression profiling and ChIP-sequencing. We identified several thousand candidate regulatory regions with altered levels of histone acetylation that were characterised by differential distribution of consensus motifs for key haematopoietic transcription factors including Gata2, Gfi1 and Sfpi1/Pu.1. In particular, downregulation of Gata2 expression was mirrored by abundant GATA motifs in regions of reduced histone acetylation suggesting an important role in leukaemogenic transcriptional reprogramming. Forced re-expression of Gata2 was not compatible with sustained growth of leukaemic cells thus suggesting a previously unrecognised role for Gata2 in downregulation during the development of AML. Additionally, large scale human AML datasets revealed significantly higher expression of GATA2 in CD34+ cells from healthy controls compared with AML blast cells. The integrated genome-scale analysis applied in this study represents a valuable and widely applicable approach to study the transcriptional control of both normal and aberrant haematopoiesis and to identify critical factors responsible for transcriptional reprogramming in human cancer.

## Introduction

Mutations in transcriptional and epigenetic regulators are a recurring theme in acute leukaemias. These mutations arise in haematopoietic stem/progenitor cells (HSPCs) and are thought to promote leukaemia by deregulating transcriptional programs controlling proliferation, differentiation and cell death [1], [2]. In about half of all acute leukaemia patients, specific chromosomal translocations are found that lead either to the creation of aberrant fusion-proteins with oncogenic potential or to the ectopic expression of proto-oncogenes [3], [4]. The majority of leukaemogenic translocations in acute myeloid leukaemia (AML) affect genes involved in transcriptional regulation or chromatin modification, thus highlighting the importance of deregulated transcriptional programs.

Mouse model systems using retroviral transduction of oncogenic fusion proteins recapitulate many aspects of the human disease and therefore represent valuable tools to dissect the molecular mechanisms causing AML [5]. *Mixed Lineage Leukaemia* (MLL) and *Monocytic Leukaemia Zinc Finger* (MOZ) fusion proteins have both been shown to subvert HSPCs into AML leukaemia cells in retroviral transplant mouse models [6], [7], [8]. Both proteins interact with the cellular epigenetic machinery, conferring either histone methyl transferase [9] or histone acetyl transferase activity [10]. MLL- and MOZ-fusion proteins are both thought to promote the leukaemic phenotype at least in part by mediating ectopic expression of abdominal *HoxA-*genes [11], [12], [13], [14], [15]. MLL fusion proteins are considered potent oncogenes and MLL-ENL causes leukaemia with relatively short latency in retroviral bone marrow transplant models [6]. AML caused by MOZ-TIF2 is also associated with a poor prognosis; however, the retroviral models have a longer latency [7] suggesting some differences within the mechanisms that drive transcriptional reprogramming.

Although, much has been learnt about the role of single transcription factors in both normal haematopoiesis and leukaemogenesis [16], [17], only the recent advent of array- and sequencing-based high-throughput technologies has enabled functional investigations on a genome-wide scale. For example, binding sites have been mapped for the stem cell regulator Scl in normal HSPCs [18] as well as the PML-RARA oncoprotein in promyelocytic leukaemia cells [19]. However, to improve our understanding of how specific leukaemogenic mutations corrupt entire transcriptional programmes, genome-wide studies need to be applied to developmental time-courses of leukaemogenesis. Of note, retroviral transduction models generate leukaemias with essentially identical immunophenotypes following initial transduction of either haematopoietic stem-cells (HSCs), common myeloid progenitors (CMPs) or granulocytic/monocytic-restricted progenitors (GMPs) [6]. This suggests that genome-wide comparisons of gene expression and ChIP-sequencing profiles between normal HSPCs and leukaemic cells might provide novel hypotheses about globally acting processes that underlie leukaemogenic transcriptional reprogramming.

In this study, we used this approach to assess and compare transcriptional reprogramming in two leukaemia progression models, based on established MLL-ENL [6] and MOZ-TIF2 [7], [20] AML mouse model systems. The experiments were designed to allow us to answer the following questions: (i) To what extent are leukaemia initiation programmes induced by MLL-ENL and MOZ-TIF2 distinct? (ii) Is progression to frank leukaemia for both oncogenes accompanied by convergence towards a shared transcriptional program? (iii) What are the advantages of combining profiling for gene-expression with ChIP-Seq compared to gene-expression alone? (iv) Does histone acetylation ChIP-Seq represent a useful tool to reliably identify alterations in candidate regulatory regions associated with transcriptional reprogramming at genome-wide scale? (v) Does bioinformatic motif analysis of altered candidate regulatory regions represent a potential avenue into identifying the regulatory processes that underlie transcriptional reprogramming?

## Results

### Mouse model systems to study transcriptional reprogramming during AML development

To study the genome-wide dynamics of leukaemogenic transcriptional reprogramming, we used two established transplantable AML mouse models based on retroviral transduction of normal bone-marrow progenitor cells with MLL-ENL or MOZ-TIF2 fusion proteins, respectively. Both mouse models are characterized by an initially longer latency, which is rapidly shortened following re-transplantation of the resultant AML-cells into secondary and tertiary recipients [6], [7]. Importantly, primary transduced pre-leukaemic cells can be cultured *in vitro* as interleukin 3 (IL3) dependent cell lines. These *in vitro* cultured cells maintain the initial long-latency when transplanted into irradiated recipients and thus represent a surrogate model for the pre-leukaemic “initiation” phase of AML [7]. By contrast, frank leukaemic cells maintain their short latency, even if exposed to periods of IL3-culturing prior to transplantation [7] which allowed us to standardise sampling conditions by overnight culture in IL3.

**Figure 1:**
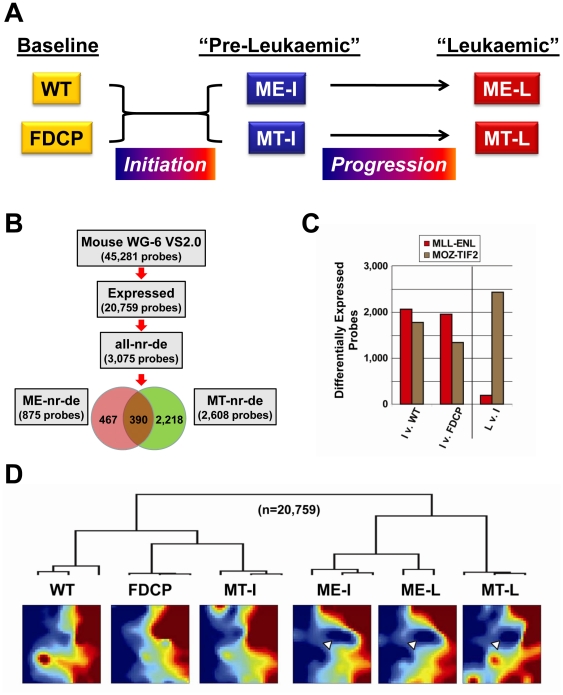
Gene-expression dynamics during MLL-ENL and MOZ-TIF2 mediated reprogramming. **A**). Diagram outlining how samples collected for expression profiling and ChIP-sequencing constitute a leukaemia progression model. **B**) Flow chart of gene-expression analysis. 20,759 of the 45,281 probes represented on the array (45.8%) were found to be expressed in at least one sample (detection p-value >0.01). Differential expression analysis was performed for six representative pair-wise comparisons, as summarized in Table S1 in Supporting Information S1. Non-redundant, differentially expressed probes (nr-de) were determined as outlined in Figure S2 in Supporting Information S1: 857 probes for MLL-ENL (ME-nr-de probes) and 2,608 for MOZ-TIF2 (MT-nr-de probes) corresponded to 3,075 non-redundant probes differentially expressed in at least one of the transitions (all-nr-de probes). **C**) Bar-charts of differentially expressed probes in six relevant pair-wise comparisons. Y-axis shows the total number of differentially expressed probes, as outlined in Table S1 in Supporting Information S1, for the “Initiation” (WT/FDCP vs ME-I/MT-I) and the “Progression” to overt leukaemia (ME-I/MT-I vs ME-L/MT-L). **D**) Unsupervised hierarchical clustering (UHC) correlates with *Gene Expression Dynamics Inspector* (GEDI) maps. All 20,759 expressed probes were clustered, as shown in the dendrogram on the top of the figure, and dynamic expression changes visualized with GEDI maps. Mean expression values of probes with similar dynamic patterns are condensed by self organizing maps in expressed (red) and repressed (blue) tiles and proximity of adjacent tiles indicates similar dynamics [60]. UHC and GEDI identified a ‘non-leukaemic’ cluster, containing the WT, FDCP and MT-I samples, and a ‘leukaemic’ cluster, comprising the ME-I, ME-L and the MT-L samples. A group of co-ordinately downregulated probes seen in the ME-I, ME-L and MT-L samples is indicated by white arrowheads.

To serve as baseline comparators for our analysis of leukaemia progression, we elected to sample two controls: (i) the lineage negative/c-kit positive (lin^-^/kit^+^) compartment of wild-type bone-marrow mononuclear cells (WT) representing the target cells transduced by the leukaemogenic retroviruses [6], and (ii) the *Factor-Dependent Cells Patterson-Mix* (FDCP-mix) cell-line, a non-transformed IL3 dependent murine progenitor cell-line, capable of haematopoietic multi-lineage differentiation and, importantly, lacking leukaemogenic potential [21]. We reasoned that use of the FDCP-mix cell-line as an additional baseline control would allow us to correct for expression changes associated with *in vitro* culture in IL3 used for “pre-leukaemic” and “leukaemic” cells. As summarized in Figure 1A, transcriptional programmes were therefore monitored at three different time points: at ‘baseline’ (for WT and FDCP-mix), following ‘initiation’ (ME-I and MT-I) and after ‘progression to the frank leukaemic state (ME-L and MT-L).

### Pre-leukaemic MOZ-TIF2 gene-expression profiles cluster with non-leukaemic, whereas MLL-ENL is more similar to leukaemic samples

To monitor global expression changes during leukaemia progression for both MLL-ENL and MOZ-TIF1, we performed gene expression profiling for three biological replicates each of the lin^-^/kit^+^ bone marrow (WT), FDCP-mix (FDCP), MLL-ENL initiation (ME-I), MOZ-TIF2 initiation (MT-I), MLL-ENL progression (ME-L) and MOZ-TIF2 progression (MT-L) samples. 20,759 of the 45,281 probes present on the array were expressed in at least one of our samples (Figure 1B). As expected, unsupervised hierarchical clustering and *Gene Expression Dynamics Inspector* (GEDI) maps of all expressed transcripts showed a clear separation of the two baselines and the two leukaemia samples (Figure 1D). Interestingly though, the gene-expression profile of MOZ-TIF2-inititation (MT-I) was closer to the non-leukaemic baseline samples WT and FDCP, whereas MLL-ENL-initiation (ME-I) was highly similar to the leukaemic samples MLL-ENL-L (ME-L) and MOZ-TIF2-L (MT-L). Further analysis of differential expression in six relevant pair-wise comparisons (summarised in Table S1 in Supporting Information S1) revealed more changes upon initiation but less at progression in MLL-ENL, compared to MOZ-TIF2 (Figure 1C), consistent with the differential clustering of the two pre-leukaemic samples.

To validate *in silico* the biological significance of the leukaemic clustering of ME-I, ME-L and MT-L, we performed *Gene Set Enrichment Analysis* (GSEA), based on a comparison with the two baselines (WT and FDCP-mix). Significant enrichments (FDR <10%) were found for curated gene-sets known to be associated with leukaemogenesis, such as up-regulated in HoxA9 and MLL-fusions or modified by *Retinoid Acid Receptor Alpha* (RARA) [22], [23], [24] (Figure S1 A,B and C in Supporting Information S1). Taken together, comprehensive gene-expression profiling analysis implies that leukaemogenic transcriptional reprogramming occurs faster with MLL-ENL than MOZ-TIF2. Moreover, while the transcriptional status of the ‘initiation’ phase is distinct for the two different oncogenes, leukaemic progression of MOZ-TIF2 samples results in a transcriptional profile much closer to the MLL-ENL samples.

### Transcriptional downregulation is a common feature of MLL-ENL and MOZ-TIF2 leukaemia models

Both fusion-proteins MLL-ENL and MOZ-TIF2 are thought to induce leukaemogenic transformation at least in part by activating abdominal *HoxA*-cluster genes. However, delayed acquisition of a full leukaemic expression profile seen with MOZ-TIF2 suggests that additional down-stream effectors/collaborators are required and occur early or late with the MLL-ENL and MOZ-TIF2 oncogenes, respectively. Of note, simultaneous availability of timecourse datasets for both MLL-ENL and MOZ-TIF2 provided an opportunity to identify some of these putative effectors by intersecting the differentially expressed genes of the “baseline”/“initiation” comparison for MLL-ENL with the “initiation”/“progression” comparison for MOZ-TIF2. This allowed us to identify 49 upregulated and 111 downregulated probes (corresponding to 40 and 88 genes), which represent to a novel candidate gene set associated with development of full leukaemia in these two AML mouse models (Figures S3, S4 and Table S2 in Supporting Information S1).

Common downregulation in the ME-I, ME-L and MT-L samples was also readily observed in the GEDI maps (see arrowheads in Figure 1D). 17 out of 23 genes in the repressed GEDI region overlapped with the shared repressed gene-set derived from the analysis of differentially expressed genes, thus underlining the consistency of our results using two independent bioinformatic approaches. The commonly activated and repressed probes, as well as probes derived from the shared repressed GEDI region are displayed in clustered heat-maps (Figure S4 in Supporting Information S1) and listed as 40, 88 and 23 unique genes, respectively (Table S2 in Supporting Information S1). Interestingly, key haematopoietic transcription factors such as Gata2, Gfi1b and Zfpm1/Fog, were found in this commonly repressed gene-set. In summary, global analysis provided evidence that transcriptional downregulation is a common feature associated with full leukaemic transition in both AML mouse models.

### Dynamic transcriptional regulation of hematopoietic stem/progenitor cell transcription factors during leukaemogenic progression

**Figure 2:**
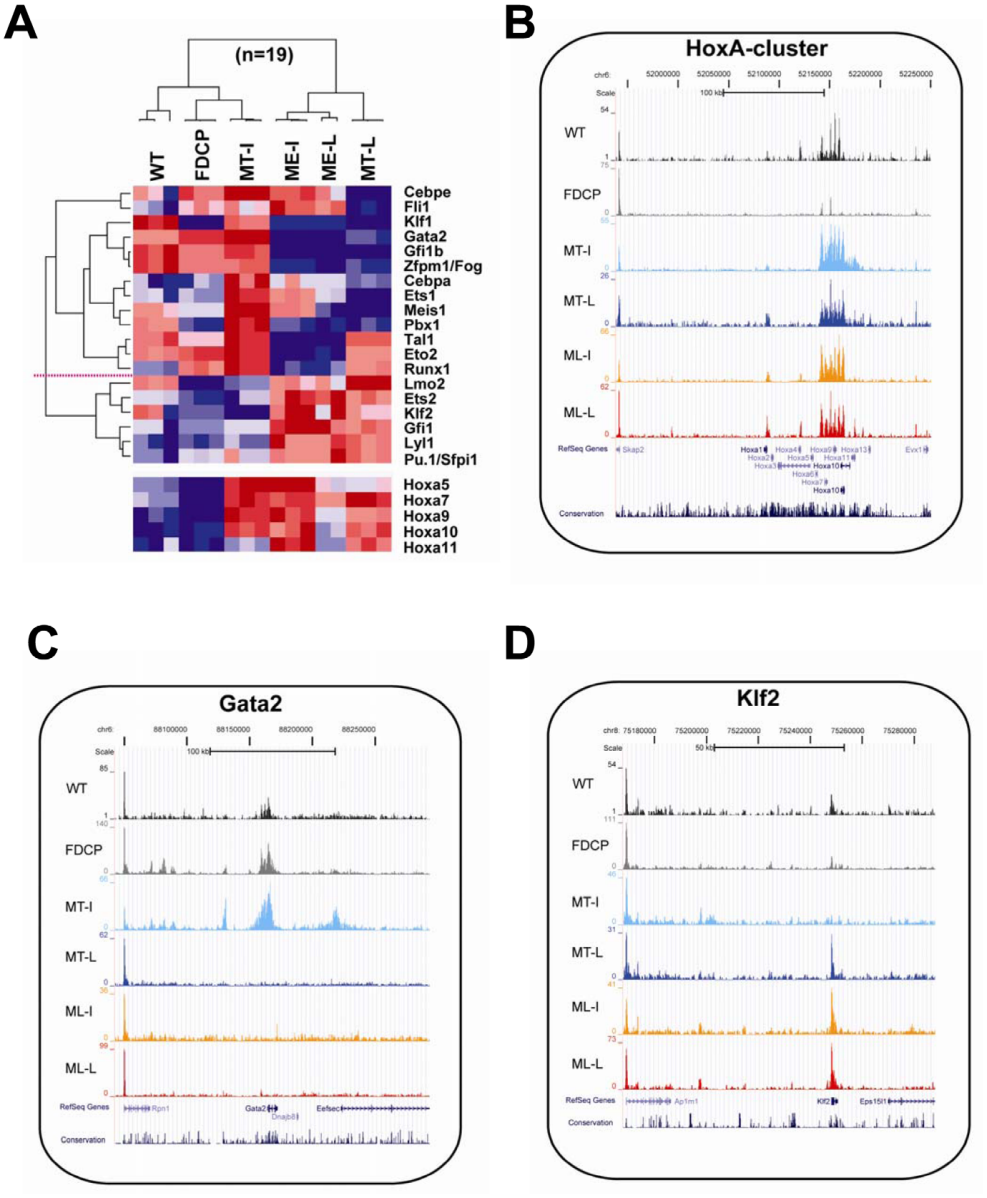
Dynamic changes of HSPC transcription factors during MLL-ENL and MOZ-TIF2 induced reprogramming. **A**) Hierarchical clustering of 19 dynamically expressed HSPC transcription factors. For comparison, dynamic expression changes of 5 abdominal *HoxA*-cluster genes are depicted as a heatmap at the bottom of the figure but were not included for the clustering analysis. Clusters of repression and activation could be distinguished and are separated by a horizontal dotted red line. **B**) Dynamic changes of histone H3K9 acetylation (H3K9ac) at the *HoxA*-cluster gene-locus. ChIP-Seq traces are displayed on the UCSC genome browser for one representative biological replicate of the six conditions under investigation (WT, FDCP, MT-I, MT-L, ME-I, ME-L). **C**) Dynamic changes of H3K9ac at the *Gata2* gene-locus. Down-regulation of Gata2 expression is paralleled by reduced acetylation marks. **D**) Dynamic changes of H3K9ac at the *Klf2* gene-locus. Up-regulation of Klf2 expression is paralleled by increased acetylation marks.

Our finding of downregulated expression of Gata2, Gfi1b and Zfpm1/Fog during leukaemic progression prompted us to investigate in more detail transcription factor gene expression patterns to identify candidate regulators of leukaemogenic transcriptional reprogramming. 121 genes functionally annotated as DNA-binding transcription factors were differentially expressed in comparisons involving MLL-ENL and/or MOZ-TIF2 time courses. These included several *HoxA* genes as well as 19 transcription factors known to function as regulators of HSPCs [25]. Unsupervised hierarchical clustering using these 19 genes generated the same partition observed above into ‘non-leukaemic’ and ‘leukaemic’ clusters with HSPC-TFs subdivided into gene-clusters of repression and activation (Figure 2A). The repression cluster could be further separated into genes repressed in both MLL-ENL and MOZ-TIF2 after progression to frank leukaemia (Klf1, Gata2, Gfi1b, Zfpm1/Fog), repression in MOZ-TIF2 only (Cebpe, Fli1), repression in MLL-ENL only (Tal1, Eto2, Runx1) and more variable patterns (Cebpa, Ets1, Meis1, Pbx1). The cluster of activation (Lmo2, Ets2, Klf2, Gfi1, Lyl1, Sfpi1/Pu.1) showed fairly homogenous activation separating the WT/FDCP/MT-I from the ME-I/ME-L/MT-L samples.

Having generated genome-wide histone acetylation datasets by ChIP-Seq in parallel to our gene-expression profiles, we next investigated whether alterations in steady state mRNA levels were accompanied by corresponding changes in levels of histone acetylation. As we and others have previously reported, cell-type specific regulatory regions can be identified by differential enrichment of H3K9ac [26], [27]. Activation of the abdominal *HoxA*-cluster genes is known to contribute to the leukaemogenic phenotype in our AML models. Increased expression of *HoxA* genes was paralleled by increased H3K9ac marks as shown in Figure 2B. Beyond the specific example of the *HoxA* locus, dynamic changes of histone acetylation closely followed the dynamics of transcriptional changes (see Figures S5, S6 and S7 in Supporting Information S1 for example plots of HSPC-TF loci). Changing levels of acetylation marks were not only seen in promoter-regions but also observed at known and potentially new distal cis-regulatory elements, as exemplified for Gata2 and Klf2 in Figure 2C and 2D. Taken together, we hypothesize that leukaemogenic progression in MLL-ENL and MOZ-TIF2 AML models is associated with altered expression of HSPC-TFs which, in turn, is accompanied by corresponding changes in H3K9ac profiles at their candidate regulatory regions.

### Over-representation of consensus binding motifs for differentially expressed HSPC transcription factors within the most variable H3K9ac candidate regulatory regions

**Figure 3:**
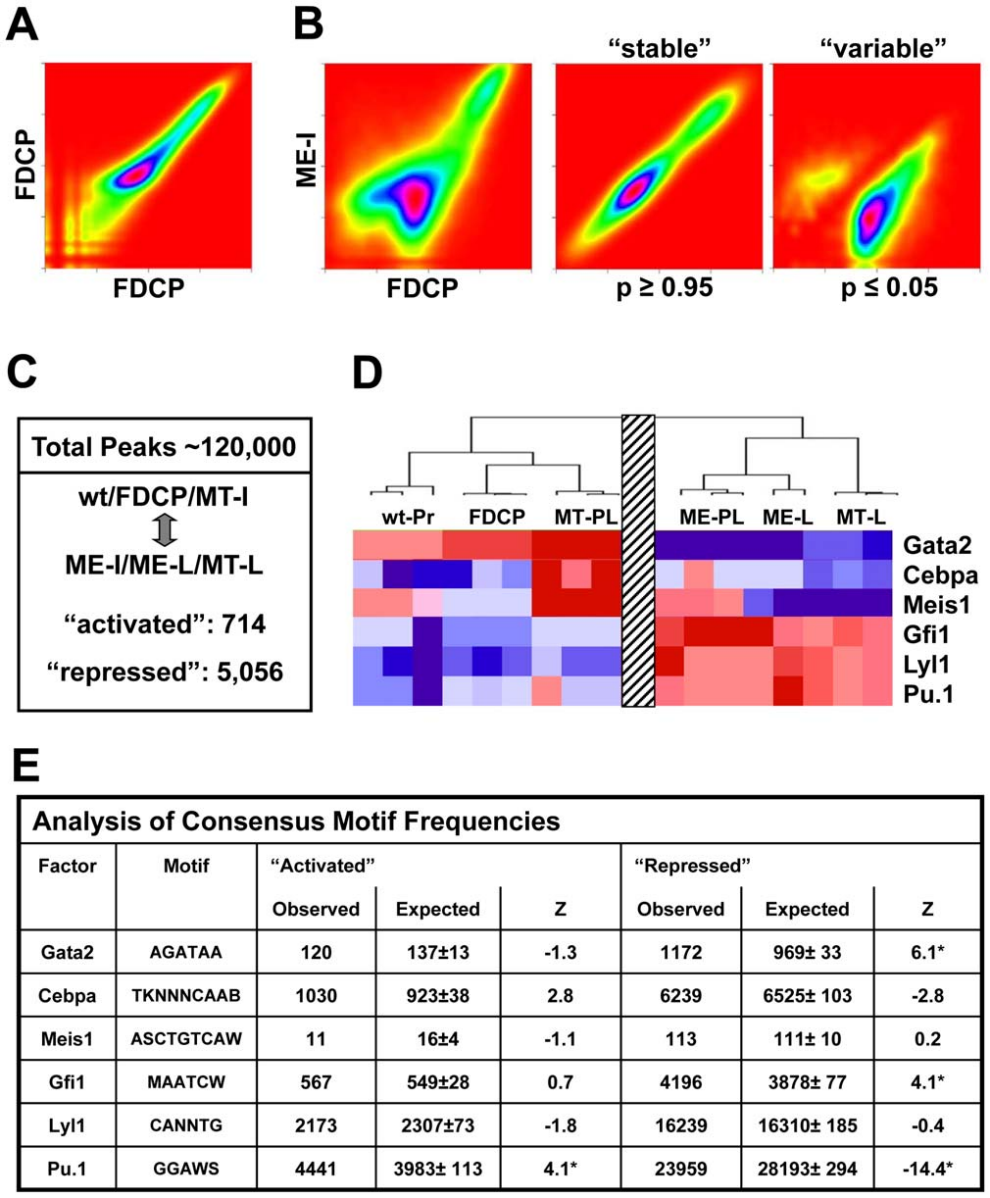
Over-representation of consensus binding motifs from differentially expressed HSPC transcription factors within most variable H3K9ac candidate regulatory regions. **A**) Kernel Density Estimation Plots (heat-maps) of relative H3K9ac peak-scores from biological replicates used for analysis of leukaemogenic progression in MLL-ENL. Peak scores are depicted as colour-coded heat-maps for the two FDCP biological replicates. Most datapoints lie on a diagonal suggesting good correlation between biological replicates. **B**) Heat-maps of relative H3K9ac peak-scores for the FDCP/ME-I ‘Initiation’ comparison as well as depiction of “stable” and “variable” regions (T-test: p≤0.05). Variable regions were clearly divided into enriched (above 45° axis) and deprived (below 45° axis) regions. **C**) Summary of dynamically changing H3K9ac candidate regulatory regions for the wt/FDCP/MT-I v. ME-I/ME-L/MT-L comparison. **D**) Expression profile for selected HSPC TFs across the wt/FDCP/MT-I v. ME-I/ME-L/MT-L comparison (derived from Fig. 2A). **E**) Observed and expected frequencies of 6 consensus-motifs in most variable H3K9ac candidate regulatory regions from C). Significantly increased and reduced peak-regions were used for the detection of differentially distributed motifs using bootstrap analysis with 1000 sets of background sequences (see methods). Significant under/over-representation was defined by a Z-score of ≤−3 or ≥+3 (indicated by a *).

We next asked whether global motif content analysis of differentially acetylated regions would allow us to draw conclusions about which transcription factors might be relevant for genome-wide transcriptional reprogramming. To this end, peaks of enriched H3K9ac were identified for all 12 ChIP-Seq samples (2 biological replicates for each of our samples) resulting in a total number of 128,354 candidate regulatory regions that were identified in at least one sample. This is in line with the approximately 100,000 regions of open chromatin thought to be characteristic for mammalian cell types [28]. We next determined the relative number of CHIP-Seq reads for each of these 128,354 regions in each sample, which demonstrated high reproducibility of biological replicates (see Figure 3A). By contrast, when comparing samples corresponding to different stages such as baseline and leukaemia initiation stages (FDCP v. ME-I), substantial numbers of peaks showed altered levels of histone acetylation (Figure 3B).

To identify those regions with statistically significant changes of histone acetylation that parallel our gene expression analysis, we partitioned the ChIP-Seq peak datasets into WT, FDCP and MT-I on the one hand and ME-I, ME-L and MT-L on the other hand, in line with the major partition identified by clustering analysis of gene expression profiles. Peak-regions with *significant* changes in H3K9ac were determined by high-dimensional T-test analysis, defining the most variable candidate regulatory regions. Of note, many more peaks showed reduced levels of histone acetylation than an increase (Fig 3C) consistent with the predominance of downregulation observed in the gene expression profiling analysis.

To identify transcription factors that may be involved in this global reorganization of chromatin, we next explored the distribution of consensus binding motifs for differentially expressed HSPC-TFs across the most variable H3K9ac peak-regions (see Material and Methods). We focused this analysis on motifs for Gata2 and Gfi1/Lyl1/Sfpi1 which were down- and up-regulated respectively across the major partition (WT/FDCP/MT-I v ME-I/ME-l/MT-L) and also included motifs for Cebpa and Meis1 as controls because expression of those two factors did not correlate with this partition (Figure 3D). When we determined the expected and observed motif-occurrences for these six factors within differentially acetylated regions defined above, consensus sites for the two controls Cebpa and Meis1 occurred in expected frequencies. By contrast, motifs for all differentially expressed factors except Lyl1 showed highly significant over and/or under-representation (Figure 3E). Of particular interest, the consensus motifs for Gata2 and Sfpi1/Pu.1 were significantly over-/under-represented respectively in regions with reduced histone acetylation, which was completely consistent with the sharp downregulation of Gata2 and upregulation Sfpi1/Pu.1 expression at this transition. Taken together, the distribution of consensus binding motifs within candidate regulatory regions with significant changes of histone acetylation revealed that differentially expressed HSPC TFs such as Gata2 and Sfpi1/Pu.1 may play a role in transcriptional reprogramming.

### Gata2 over-expression inhibits expansion of MLL-ENL transduced cells

Integrated analysis of gene-expression and ChIP-Seq profiling datasets suggested a role for Gata2 downregulation during the development of AML in our mouse model system. To identify a potential functional role for Gata2 downregulation, we re-expressed Gata2 in MLL-ENL transduced cells. Expression vectors with IRES-dependent GFP-expression were used to monitor the proportion of GFP-positive cells in competitive proliferation assays performed in liquid and semi-solid conditions. We compared the effect of the empty vector *Mscv-PIG* (PIG) with full length Gata2 (Gata2) and a Gata2 deletion mutant (ΔGata2) (see Figure S8A in Supporting Information S1), which only contains the 294 N-terminal amino-acids and thus lacks the two zinc-fingers required for DNA-binding (see Figure S8B in Supporting Information S1). Both constructs produced the expected RNA and protein products see Figure S8C, D and E in Supporting Information S1).

**Figure 4:**
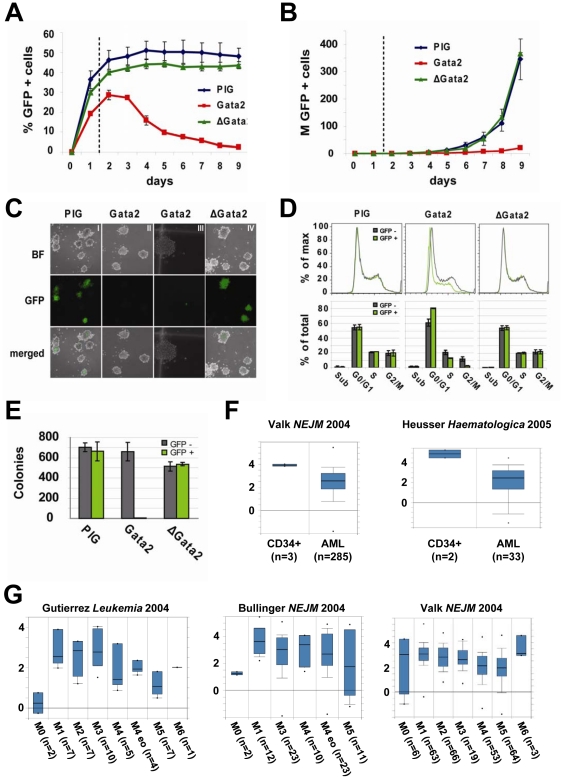
Gata2 over-expression interferes with cell-cycling and is incompatible with sustained proliferation of MLL-ENL transduced cells. **A**) Fraction of GFP-positive cells monitored in a competitive proliferation assay performed in liquid culture over a time-course of 9 days. ME-I cells were transduced with *pMSCV-Pgk-Puro-IRES-GFP* (PIG), *pMSCV-Gata2-Pgk-Puro-IRES-GFP* (Gata2) and *pMSCV-*Δ*Gata2-Pgk-Puro-IRES-GFP* (ΔGata2). Vector constructs are outlined in Figure S9A and B in Supporting Information S1. X-axis depicts days after transduction, y-axis the GFP-positive cell-fraction (% of total viable cells). GFP-positive and GFP-negative cells of each sample were analysed together without prior FACS-sorting. Results from two independent experiments (mean ± SD) are shown. Subsequent analysis (Figure 4D) was performed 36 hours after transduction (indicated by the dotted line). **B**) Growth curves show that Gata2 overexpression is incompatible with sustained proliferation of ME-I cells. X-axis depicts days after transduction, y-axis the total GFP-positive cell-number (in millions) determined from the GFP-positive fraction and the total number of viable cells. Results from two independent experiments (mean ± SD) are shown. **C**) Representative pictures of colony-formation in a competitive proliferation assay performed in semi-solid culture. ME-I cells were transduced with PIG, Gata2 and ΔGata2 and 10^4^ cells transferred to methylcellulose after 36 hours. Images were taken after 4 days under bright-field (BF) and with GFP fluorescent light (GFP) and merged with 10x magnifications (except section III, which was taken at 20x magnification). **D**) Cell-cycle analysis of GFP-positive and GFP-negative cells 36 hours after transduction. The upper panel depicts a representative cell-cycle plot of GFP-positive and GFP-negative ME-I cells after transduction with PIG, Gata2 and ΔGata2. Note that GFP-positive and GFP-negative cells of each sample were analysed together without prior FACS-sorting, providing an internal control for gating and settings of the instrument. X-axis shows PI fluorescence intensities, y-axis % of maximal counts. The lower panel depicts % of total counts in sub-G0/G1 (sub), G0/G1, S and G2/M-phase as determined by the cell-cycle plot for GFP-positive and GFP-negative cells. Results from two independent experiments (mean ± SD) demonstrated statistically significant alterations for the Gata2 transduced cells (sub, p = 0.97; G0/G1, p = 0.033; S, p = 0.068; G2M, p = 0.041; two-tailed t-test). **E**) Bar-chart of numbers of GFP-positive and GFP-negative colonies from C). Results from two independent experiments (mean ± SD) are shown. **F**) Comparison of GATA2 expression from published human AML microarray datasets performed with *Oncomine* (http://www.oncomine.org) [62]. Boxes display median expression values and contain data from the 25^th^ to 75^th^ percentiles with the bars representing the 10^th^ and 90^th^ percentiles respectively. Y-axis show log2 median centered ratios. The Valk et al dataset (GEO accession number: GSE1159) [31] shown on the left shows relatively high GATA2 expression in CD34+ control cells when compared with bone marrow samples from 285 AML-patients (probe H00625, note that 10th percentile of the CD34 samples is higher than the 90th percentile of the AML samples, thus indicating a significant difference in expression levels). The Heuser et al dataset (GEO accession number: GSE4137) [32] on the right similarly shows significantly higher levels of GATA2 in CD34+ cells from two healthy controls compared to blood and bone marrow samples from 33 AML-patients prior induction chemotherapy. **G**) Oncomine analysis of three independent gene expression datasets (GEO accession numbers: GSE1729, GSE425, GSE1159) [31], [33], [34] demonstrates that GATA2 expression levels are not higher in AML subtypes characterised by minimal differentiation (FAB AML-M0).

In liquid-culture, GFP-positive cells over-expressing Gata2 demonstrated a competitive disadvantage by comparison with the GFP-negative cell fraction and showed highly reduced growth-kinetics when compared with cells transduced with the empty vector and the ΔGata2 constructs (Figures 4A and B). In concordance with this result, Gata2 over-expressing cells displayed a near complete failure of colony formation in semi-solid conditions, whereas the number of GFP positive and negative colonies was comparable for the negative controls (Figure 4C and E). To investigate the proliferation defect mediated by Gata2 in more detail, we performed cell-cycle and apoptosis analysis 36 hours after transduction. By analyzing GFP-positive and GFP-negative cells from the same sample in parallel, we were able to perform comparative analysis both within and across different samples. Consistent with the proliferation assays, Gata2 over-expressing samples showed an increase of cells in G0/G1- and a corresponding decrease in S/G2/M-phase, compared to the internal and external controls (Figure 4D). Importantly, 293T cells showed no obvious differences in growth or appearance when transfected with the same Gata2 expression or the two control constructs (PIG and ΔGata2). We have therefore no reasons to assume that high-level expression of Gata2 is non-specifically toxic to transduced cells. Moreover, we did not observe any significant effect on apoptosis by AnnexinV staining and no increase of quiescence by Ki67-staining 36 hours after infection of MLL-ENL cells (see Figure S9 in Supporting Information S1). In summary, over-expression of Gata2 is incompatible with sustained proliferation of MLL-ENL transduced cells consistent with a model whereby downregulation of Gata2 plays a role during the early stages of leukaemogenic reprogramming.

### Downregulation of GATA2 in human AML

Any potential involvement of GATA2 downregulation in the development of haematological disorders remains as yet unclear [29], [30]. However, when we analysed published gene-expression datasets, two collections of 285 and 33 AML patients showed higher levels of *GATA2* gene-expression in CD34+ cells from healthy controls compared with AML blast cells [31], [32] (Figure 4F). Similar levels of *GATA2* were found in all subtypes of AML and analysis of two additional datasets [33], [34] demonstrated that AML with minimal differentiation (FAB AML-M0) showed the lowest levels of *GATA2* (Figure 4G). This latter observation suggests that while *GATA2* expression is downregulated during normal myeloid differentiation, low *GATA2* expression in AML cells is unlikely to be simply related those myeloid differentiation steps that can still occur in leukaemic cells as it already occurs in the phenotypically most immature AML samples. Publicly available gene-expression datasets therefore corroborate a potential role for GATA2 downregulation in the development of human AML.

## Discussion

New genome-wide experimental approaches are widely reported as the ‘next generation’ of technologies that will revolutionise our understanding of cancers such as acute leukaemia. However, this goal is substantially complicated by the fact that acute leukaemias are caused by heterogeneous genetic and epigenetic events, which can occur at multiple levels during the dynamic progression from a premalignant state to frank leukaemia. For example, while genome-wide mapping of MLL-AF4 binding events identified stem cell regulators as prevalent target genes [35], little is known about the dynamic nature of global transcriptional reprogramming required for initiation and subsequent progression to the full leukaemic phenotype. Importantly, a focus on transcriptional reprogramming has the potential to identify shared aspects of transcriptional dysregulation and thus reduce the complexity and heterogeneity of diverse oncogenic events to a small number of specific pathways for exploration as novel disease classifiers and/or potential drug targets.

Studying the initiation or early progression of diseases such as AML is not feasible in human patients. However, mouse models of AML not only represent a valuable model for end-stage disease but also possess several qualities that make them amenable for studying disease initiation and progression. Firstly, the time point of the primary hit responsible for initiation of the disease can be strictly controlled and subsequent changes monitored. Secondly, monitoring changes within AML progression models has the potential advantage to identify new effectors/collaborators, which usually remain undetectable by approaches focussing only on frank leukaemia. Thirdly, different AML mouse model systems are available, which rely on transduction of distinct oncogenes with varying latency periods, and hence provide a suitable platform to identify shared pathways of general relevance for leukaemogenesis. Mouse models therefore represent powerful tools to study mechanisms of leukaemogenesis. Nevertheless, molecular targets identified in mouse model systems need to be further validated in human cells and xenotransplantation models to demonstrate potential clinical applicability [36].

In this study, we specifically focussed on two broadly accepted AML mouse models based on transplantation of stem/progenitor cells retrovirally transduced with the oncogenic fusion-proteins MLL-ENL and MOZ-TIF2, respectively [6], [7], [20]. The cell of origin for leukaemic transformation is still a matter of ongoing debate. Our selection of lin^-^/kit^+^ as control sample is in line with current evidence where transduction of either self-renewing HSC or short-lived CMP or GMP generated leukaemic cells with a similar immunophenotype [6]. All three of these starting populations are highly enriched in our lin^-^/kit^+^ control population. Moreover, expression analysis of HSPC TFs (such as *HoxA9, Tal1, Scl, Lmo2*) and myelomonocytic markers (such as *GM-Csfr, M-Csfr and G-Csfr*) paralleled those described by Cozzio et al [6] (see Figure S10 in Supporting Information S1). This observation underlines the similarity of our model system with previously published results and further validates the suitability of the lin^-^/c-kit^+^ control population.

We and others have previously reported that promoters and regulatory elements are enriched for H3K9ac marks [26], [27]. Distinct histone marks, sometimes combined with DNA binding proteins, have been used to identify potential regulatory regions [37], [38]. However, as long as regulatory elements are incompletely identified and characterized, there is no single epigenetic mark ‘of choice’ that can be considered the most appropriate to highlight regulatory regions [39], [40]. In both AML models, we observed upregulation of abdominal *HoxA*-gene expression as expected. Moreover, this was paralleled by substantially increased levels of histone acetylation and therefore validated our experimental approach of using regions of altered histone acetylation to interrogate transcriptional control mechanisms that may underlie leukaemia development. However, downstream effectors and/or or parallel contributors other than a deregulated Hox-program are required for leukaemia development [11], [41]. The integrated genome-wide approach used here provided a potential strategy to identify some of these additional transcriptional events. Inference of transcriptional control mechanisms from microarray expression profiling data alone has been attempted in the past using bioinformatic tools that typically search promoter regions or collections of evolutionarily conserved sequence blocks for overrepresented sequence motifs. When we examined our list of differentially expressed genes using two of the most widely used tools for this type of *in silico* analysis, *oPOSSUM* [42] only identified the binding site for one of the 19 factors shown in Figure 3A (i.e. Cebpa) and *Webmotifs* [43] failed to identify any significant motifs at all (see Figure S11 in Supporting Information S1). By contrast, our direct measurements of differential levels of histone acetylation allowed us to select an experimentally informed set of candidate regulatory regions that showed differential distribution of the binding sites for 3 of the 4 differentially expressed factors including the binding site for Gata2, the potential relevance of which we went on to validate experimentally. Consistent with arguments put forward in a recent review [40], integrated genomic approaches, such as expression and ChIP-Seq profiling used here, provide a much less arbitrary way to select regions for sequence motif analysis than pure *in silico* approaches and should therefore be widely applicable to study transcriptional dysregulation in disease.

In all current AML models, leukaemic cells display a somewhat more mature surface marker phenotype compared with the cells originally transduced, suggesting that expression changes alone can not be taken as evidence for functional significance but need to be followed up with specific experimentation. Integrated expression and ChIP-Seq profiling implicated downregulation of Gata2 as an important step during MLL-ENL and MOZ-TIF2 driven leukaemogenesis. Gata2 is a major regulator of haematopoietic stem cells during ontogeny [44] and a key component of wider HSC regulatory networks [45], [46], [47]. High expression levels of Gata2 are found in HSPC and down-regulation is required during normal haematopoietic differentiation [48]. Interestingly, enforced expression of Gata2 blocks normal haematopoiesis [49] and induces quiescence in HSCs [50]. Gata2 therefore represents a major regulator of HSPC homeostasis. Our finding that downregulation of Gata2 is a common feature for two AML mouse model systems suggests that abrogation of a Gata2-dependent transcriptional program may contribute to leukaemogenic transformation which is supported further by low GATA2 expression found in large-scale human AML datasets. Acute lymphoid leukaemia patient samples show overlapping, yet on average even lower levels of GATA2 than AML (data not shown) suggesting that the incompatibility of high GATA2 expression with leukaemia development may extend beyond AML. Additionally, two GATA-2 mutations (Δ341–346 and L359V), affecting the second zinc-finger domain (see Figure S8B in Supporting Information S1) and conferring either reduced or increased transcriptional activity, respectively, have recently been described in patients progressing from chronic to acute myeloid leukaemia.[51]. These findings are in line with our conclusion that deregulated Gata2 expression is involved in leukaemogenic progression, although, the exact mechanisms remain still to be elucidated. Of note, high-level ectopic expression of Gata2 in the murine myeloid cell line 416B did not inhibit proliferation, but instead allowed the derivation of clonal cell lines with a propensity for megakaryocytic differentiation [52], thus suggesting that Gata2-mediated inhibition of proliferation is context dependent. Importantly, our observation that MLL-ENL transduced cells remain susceptible to Gata2 mediated induction of cell-cycle arrest reveals a potential Achilles heel that could be characteristic for MLL-ENL leukaemic cells and that warrants further investigation.

Taken together, gene-expression and ChIP-Seq profiling coupled with bioinformatic analysis and functional validation allowed us to identify a potentially important, yet previously unknown regulatory process, that operates during transcriptional reprogramming in two mouse models of AML. Our results suggest that down-regulation of Gata2 may contribute to leukaemogenic transformation and that reactivation of Gata2 could be a novel treatment strategy in patients with acute leukaemias. Moreover, further analysis of the transcriptional repression program associated with the leukaemic phenotype may provide new mechanistic insights into the efficacy of de-repressive epigenetic therapies such as inhibition of histone deacetylases and DNA-methyltransferases [53], [54]. Finally, the integrated genome-wide approach employed in this study should be readily adaptable to study transcriptional reprogramming in other leukaemias as well as many solid tumours.

## Materials and Methods

### Ethics statement

All animal experiments were performed under strict adherence to UK home office regulations, licensed by the UK home office under Project Licence PPL80/1900 and approved by the local Cambridge University Licence Review Committee (LRC).

### Cell samples

Wild-type bone marrow cells from Bl/6 mice were lineage depleted following red blood cell lysis and purified using Fluorescent Activated Cell Sorting (FACS) after staining with c-kit. The *Factor-Dependent Cells Patterson-Mix* (FDCP-mix) were cultured as originally described [21]. Previously described plasmids for MLL-ENL and MOZ-TIF2 fusions with *Neomycin* selection marker or *GFP* were used for *in vitro* replating experiments and *in vivo* transplantation experiments [7], [14], [55]. Retroviral transduction of murine bone marrow cells, serial replating and transplantation assays were performed as previously described [7], [14]. Factor-dependent cells capable of sustained growth in liquid medium supplemented with recombinant IL3 were generated by serial replating. ‘Leukaemic’ sample material was GFP-positive splenocytes collected from secondary recipient mice upon frank leukaemia development.

### Gene-expression and ChIP sequencing

Biotin-labelled cRNA from three biological replicates was generated from 250 ng of total RNA, hybridized onto MouseWG-6 version 2 Expression Bead Chips (Illumina, San Diego, CA) and data processed with the *lumi Bioconductor* package [56] including variance-stabilizing transformation and quantile normalization [57]. The human/mouse probesets for analysing GATA2 gene expression were H00625 and ILMN_2612283 respectively. Two biological replicates per condition were used for ChIP-Sequencing (ChIP-Seq) as previously described [18] [58] using polyclonal rabbit anti-acetyl histone H3K9 (Upstate). Unique sequencing reads were mapped to the reference genome build mm8 (February 2006), extended strand-specifically to 200 bp and displayed as density-plots using the UCSC genome-browser.

### Bioinformatic analysis

Gene expression data were deposited at the NCBI Gene Expression Omnibus (accession number GSE25539). Differentially expressed transcripts were determined using the *limma Bioconductor* package at a False Discovery Rate (FDR) of ≤0.001 [59]. Unsupervised hierarchical clustering was performed using the *GenePattern* platform (genepattern.broad.mit.edu) by applying complete-linkage with Euclidian distances for samples and Pearson correlations for probes. *Gene Expression Dynamics Inspector* (GEDI) was used to visualize expression dynamics[60], *Gene Set Enrichment Analysis* (GSEA) to identify enrichment for curated gene-sets (www.broadinstitute.org/gsea/) [61] and *Oncomine* (www.oncomine.org) to analyse Gata2 expression in leukaemia datasets [62].

ChIP-Seq peaks were identified using *Findpeaks 3.1* at FDR ≤0.05 [63] and used to define 400 bp-regions centered around the highest point of significant peaks. Non-redundant regions were scored by counting all 200 bp-extended reads within the region and normalized to the total number of sequence-reads of the corresponding sample. Significant changes of histone acetylation between pair-wise comparisons of different conditions were identified with the *Cyber-T-test* R-module by using all biological replicates [64] and graphically displayed in kernel density estimation plots. Significance thresholds were set at p<0.05 for the most variable regions, which were further subdivided into enriched and deprived regions. Motif analysis was performed using TFBSsearch [65] with consensus motifs derived from the UniProbe and Jaspar databases. Expected frequencies were obtained by applying a standard boot-strapping procedure (1000 random samples) from the total number of peak-regions, providing a random distribution of expected occurrences for each consensus-motif for subsequent Z-score analysis.

### Re-transduction of pre-leukaemic cells and competitive proliferation assays in liquid or semi-solid culture

Full length *Gata2* cDNA (MSCV-Gata2) and a 3′-deletion (MSCV-ΔGata2; 294 N-terminal amino-acids) were inserted into *pMSCV-Pgk-Puro-IRES-GFP* (MSCV-PIG). Ecotropic viral supernatants were produced and MLL-ENL pre-leukaemic cells retransduced as previously described [7]. Competitive proliferation assays in liquid culture were performed by monitoring the GFP-positive cell-fraction of the re-transduced MLL-ENL cells by FACS-analysis over a 9-day time-course. Semi-solid assays were performed by seeding 1×10^4^ retransduced MLL-ENL cells in *Methocult GF M3434* (StemCell Technologies) 36 hours after infection. GFP-positive and negative colonies were counted after four days with a *Zeiss inverted 200M* fluorescent microscope (Carls Zeiss Ltd, Hertfordshire, UK), representative pictures taken with a *Hamamatsu Orca ER* camera (Hamamatsu Photonics Ltd, Hertfordshire, UK) and images processed with *Improvision Openlab* software 5.5.0 (Perkin Elmer, Waltham, Massachusetts).

### Cell-Cycle and morphological analysis, AnnexinV and Ki-67 staining

Cell cycle analysis and AnnexinV staining were performed as previously described [66] and without prior sorting. FACS-analysis was run in parallel for the GFP- positive and -negative cells and data visualized by *FlowJo* (Tree Star Inc., Ashland, OR). Ki-67 staining was performed 36 hours after infection on sorted, GFP-positive and -negative cells following the manufacturers' instructions (BD, 556026).

## Supporting Information

Supporting Information S1 Supporting materials for the manuscript “Genome-wide Analysis of Transcriptional Reprogramming in Mouse Models of Acute Myeloid Leukaemia” by Bonadies et al, including the following: Supporting Figures S1 to S11 and Supporting Tables S1 and S2.
(PDF)
