# Supplementary material for: Genome-Wide Analysis of Transcriptional Reprogramming in Mouse Models of Acute Myeloid Leukaemia
Source: PLoS One. 2011 Jan 28;6(1):e16330. doi: 10.1371/journal.pone.0016330 (PMC3030562; doi:10.1371/journal.pone.0016330)
Supplement: Supporting Information S1 — Supporting materials for the manuscript “Genome-wide Analysis of Transcriptional Reprogramming in Mouse Models of Acute Myeloid Leukaemia” by Bonadies et al, including the following: Supporting Figures S1 to S11 and Supporting Tables S1 and S2. (PDF) [file pone.0016330.s001.pdf]

A

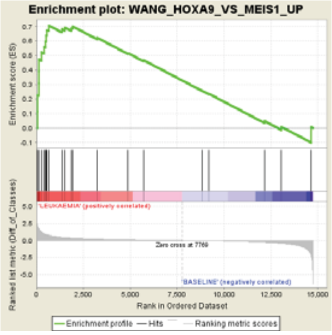

GENE SYMBOL	GENE_TITLE
MTUS1	mitochondrial tumor suppressor 1
CSF2RA	colony stimulating factor 2 receptor, alpha, low-affinity (granulocyte-macrophage)
RHOJ	ras homolog gene family, member J
ELA2	elastase 2, neutrophil
SORT1	sortilin 1
PDE2A	phosphodiesterase 2A, cGMP-stimulated
PRTN3	proteinase 3 (serine proteinase, neutrophil, Wegener granulomatosis autoantigen)
TLR4	toll-like receptor 4
IGSF6	immunoglobulin superfamily, member 6
CD177	CD177 molecule
ALAS1	aminolevulinate, delta-, synthase 1

B

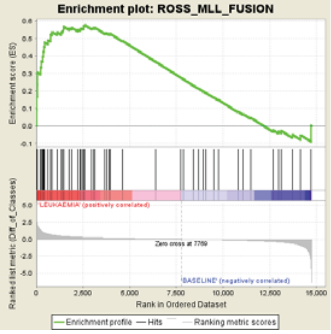

GENE SYMBOL	GENE_TITLE
ALDH3B1	aldehyde dehydrogenase 3 family, member B1
HOXA5	homeobox A5
HOXA9	homeobox A9
SIRPA	signal-regulatory protein alpha
GAS7	growth arrest-specific 7
RNPEP	arginyl aminopeptidase (aminopeptidase B)
HK3	hexokinase 3 (white cell)
MFSD1	major facilitator superfamily domain containing 1
APOC2	apolipoprotein C-II
LY86	lymphocyte antigen 86
ANXA5	annexin A5
TDRD7	tudor domain containing 7
CCL5	chemokine (C-C motif) ligand 5
MBNL1	muscleblind-like (Drosophila)
APH1B	anterior pharynx defective 1 homolog B (C. elegans)
FBP1	fructose-1,6-bisphosphatase 1
ALDH3A2	aldehyde dehydrogenase 3 family, member A2
FES	feline sarcoma oncogene
SPG21	spastic paraplegia 21, maspardin (autosomal recessive, Mast syndrome)
LRMP	lymphoid-restricted membrane protein
GGA2	golgi associated, gamma adaptin ear containing, ARF binding protein 2
TPP1	tripeptidyl peptidase I
AK2	adenylate kinase 2
CAPG	capping protein (actin filament), gelsolin-like
PYCARD	PYD and CARD domain containing
CPM	carboxypeptidase M
PBX3	pre-B-cell leukemia transcription factor 3
MRPL33	mitochondrial ribosomal protein L33

C

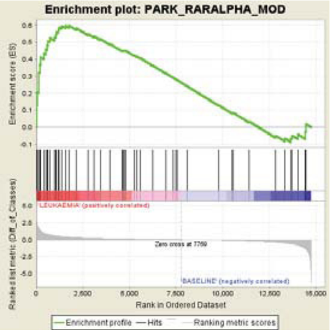

GENE SYMBOL	GENE_TITLE
APP	amyloid beta (A4) precursor protein (peptidase nexin-II, Alzheimer disease)
SIRPA	signal-regulatory protein alpha
ELA2	elastase 2, neutrophil
LYZ	lysozyme (renal amyloidosis)
GPNMB	glycoprotein (transmembrane) nmb
NEU1	sialidase 1 (lysosomal sialidase)
PRTN3	proteinase 3 (serine proteinase, neutrophil, Wegener granulomatosis autoantigen)
SRF	serum response factor (c-fos serum response element-binding transcription factor)
RAB27A	RAB27A, member RAS oncogene family
SERPINB2	serpin peptidase inhibitor, clade B (ovalbumin), member 2
ASAH1	N-acylsphingosine amidohydrolase (acid ceramidase) 1
SLC29A1	solute carrier family 29 (nucleoside transporters), member 1
GAL	galanin
COL4A2	collagen, type IV, alpha 2
CORO1A	coronin, actin binding protein, 1A
RAB31	RAB31, member RAS oncogene family
MPO	myeloperoxidase
TMSB10	thymosin, beta 10
ZYX	zyxin

Supporting Figure S1 *Gene Set Enrichment Analysis* (GSEA) of leukaemic (ME-I, ME-L, MT-I) vs baseline (WT, FDCP-mix) sample-sets (Mootha et al., 2003; Subramanian et al., 2005). Three representative plots of curated gene-sets enriched with FDR <10% in the leukaemia-sample class and a list of their corresponding core enriched genes are depicted. GSEA was run at 1000 permutations and default settings were changed, owing to the low sample number and the log transformed expression data, to '*gene_set*' as permutation type and '*Diff_of_Classes*' as metric for ranking. Enrichment for *WANG_HOXA9_VS_MEIS1_UP* (A), *ROSS_MLL_FUSION* (B) and *PARK_RARALPHA_MOD* (C) validated the 'leukaemic' cluster defined in our AML progression model.

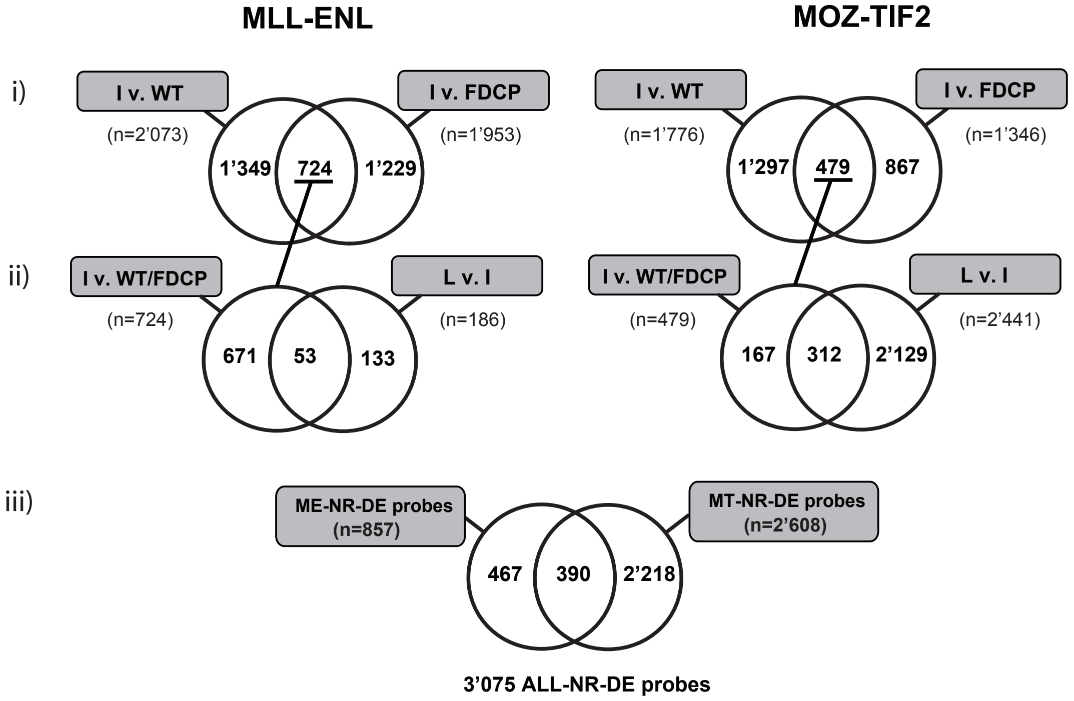

Supporting Figure S2 Strategy for determination of non-redundant, differentially expressed probes (NR-DE probes). Differentially expressed probes, generated from a representative analysis of six pair-wise comparisons as outlined in Table S1 in Supporting Information S1, were further processed in three steps: i) Interception between the two baseline controls WT and FDCP-mix provided 724 and 479 shared differentially expressed probes for MLL-ENL and MOZ-TIF2, respectively. ii) Those intersected probes were further used to define non-redundant differentially expressed probes for MLL-ENL (n = 857) or MOZ-TIF2 (n = 2'608), respectively (ME- and MT-NR-DE), considering unique probes at "baseline" and "progression" to frank leukaemia. iii) The final set of overall non-redundant, differentially expressed probes for both fusion proteins was defined by intersecting ME- and MT-NR-DE probes (n = 3'075, ALL-NR-DE probes).

Bonadies et al, Suppl Figure 2

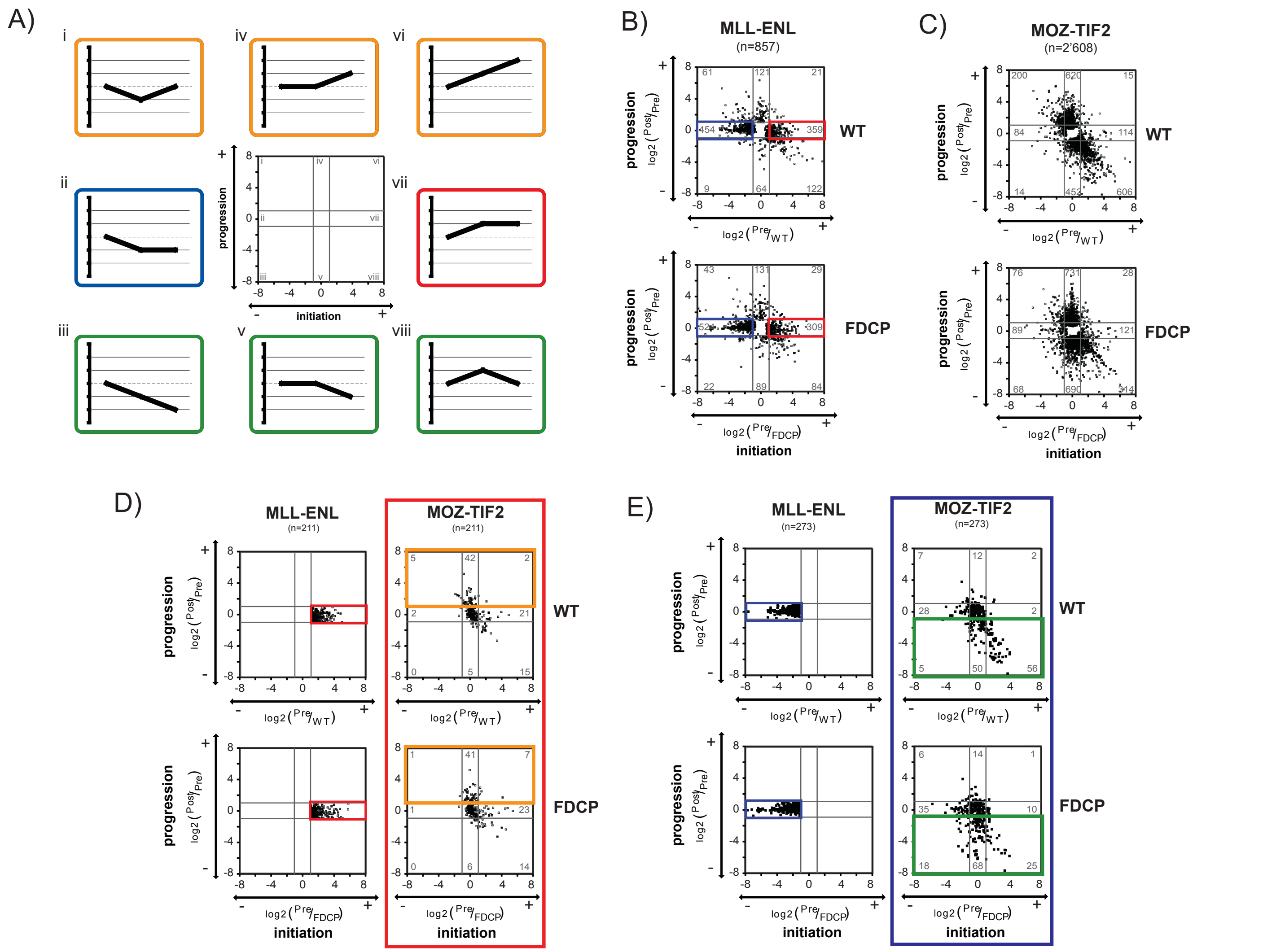

Supporting Figure S3 **A)** Graphical representation of dynamic changes in gene-expression at "initiation" and "progression" to frank leukaemia. The x-axis depicts log2 transformed ratios of mean gene expression values from "pre-leukaemic" vs "baseline" states, either for WT or FDCP-mix used as baseline (changes at "initiation"). Positive values represent up-regulated at "initiation", negative values the opposite. The y-axis shows the same for "leukaemic" vs "pre-leukaemic" states (changes at "progression" to full blown leukaemia) and positive values represent upregulated at "progression", negative values the opposite. Eight areas can be defined by using thresholds of 2-fold positive or negative changes (±1xlog2) and numbers of probes localized in the corresponding area are shown in light gray in the original graphs. Pattern of dynamic changes from "initiation" to "progression" are visualized in graphs i-viii. Probes in area i-iii are down-regulated, whereas probes in vi-viii are up-regulated at "initiation". Probes in area ii (blue) are exclusively down-regulated and probes in vii (red) are exclusively up-regulated at "initiation". Probes in areas i, iv, vi are up-regulated (orange), and probes in iii, v, viii are down-regulated at "progression" to full blown leukaemia (green). All dynamic changes at "initiation" and "progression" can be concisely visualised in this two-dimensional plot. **B)** Two-dimensional dot-plots of dynamic changes in gene-expression for the 857 ME-NR-DE probes in MLL-ENL. Blue boxes highlight the area of repression only at "initiation" (area ii), whereas the red boxes show the area of activation only at "initiation" (area vii). Note that the distribution of dot-densities in MLL-ENL is completely different compared to MOZ-TIF2 and follows a more horizontal pattern, which implies that many changes occur early at "initiation" with MLL-ENL. **C)** Two-dimensional dot-plots of dynamic changes of gene-expression for the 2'608 MT-NR-DE probes in MOZ-TIF2. The distribution of dot-densities follows a more vertical pattern, in difference to MLL-ENL. **D)** Two-dimensional dot-plots for identification of shared activated probes at leukaemic transition of MLL-ENL and MOZ-TIF2. Note that leukaemic transition occurs at different stages; at "initiation" for MLL-ENL but at "progression" for MOZ-TIF2 (see clustering and GEDI-maps in Figure 2B). The 211 probes depicted in this plots derived from intersection of the 359 and 309 probes in the red boxes shown in Figure S3B in Supporting Information S1, which represent the intersection for WT and FDCP-mix of exclusively up-regulated probes in MLL-ENL at "initiation". Those 211 probes are plotted for MOZ-TIF2 (big red box) and lead to the identification of 49 probes (22.2%), which are upregulated at "progression" in MOZ-TIF2 and highlighted in orange boxes for WT and FDCPmix baseline, respectively. These probes represent the **shared activated probes** at leukaemic transition of MLL-ENL and MOZ-TIF2. **E)** Two-dimensional dot-plots for identification of shared repressed probes at leukaemic transition of MLL-ENL and MOZ-TIF2. The 273 probes depicted in this plots derived from intersection the 454 and 524 probes in the blue boxes shown in Figure S3B in Supporting Information S1, which represent the intersection for WT and FDCP-mix of exclusively down-regulated probes in MLL-ENL at "initiation". Again, those 211 probes are plotted for MOZ-TIF2 (big blue box) and lead to the identification of 111 probes (40.7%), which are down-regulated at "progression" in MOZ-TOF2 and evidenced in green boxes for WT and FDCP-mix baseline, respectively. These probes represent the **shared repressed probes** at leukaemic transition of MLL-ENL and MOZ-TIF2.

Bonadies et al, Suppl. Figure 3

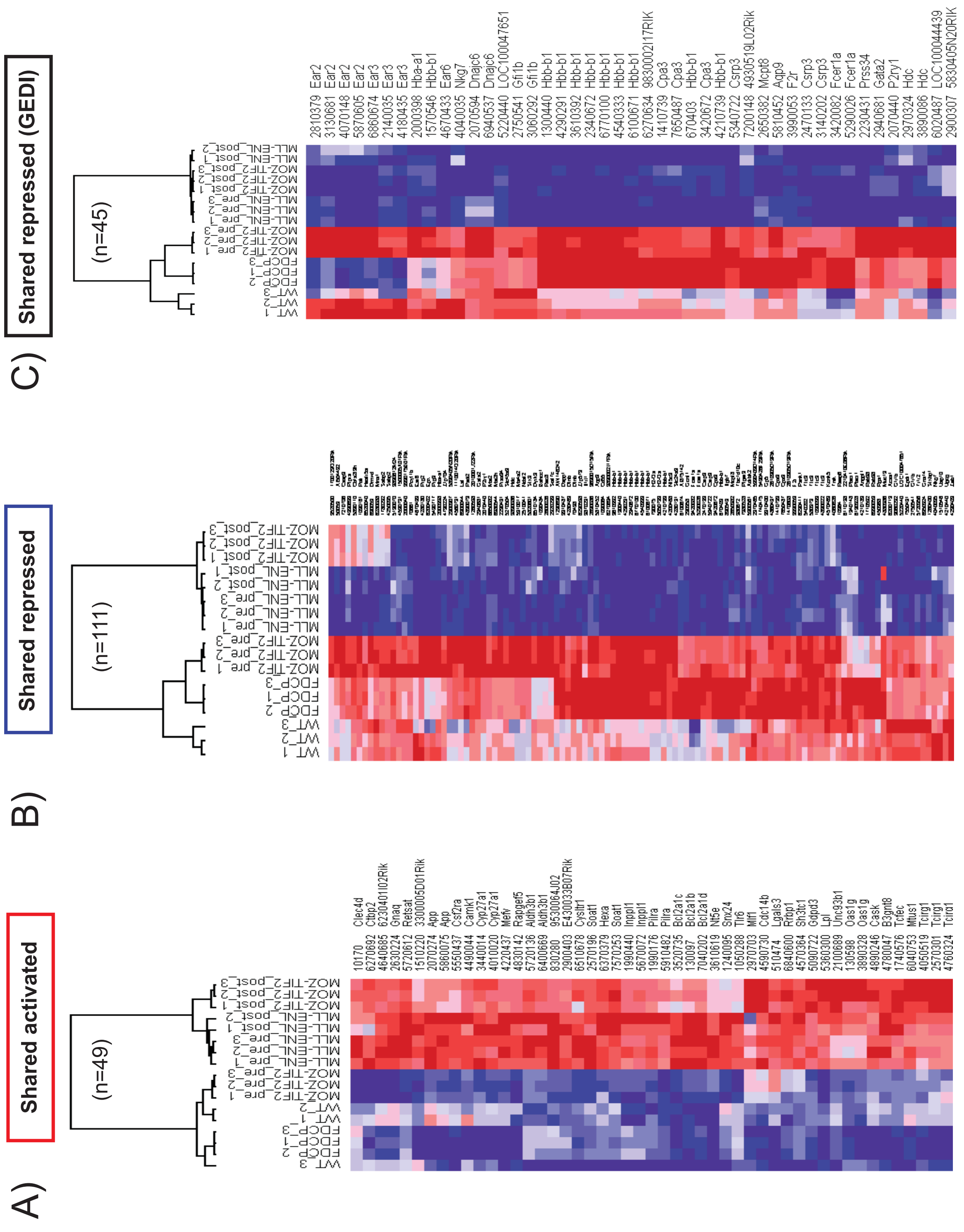

Supporting Figure S4 Gene-expression profile of shared probes at leukaemic transition of MLL-ENL and MOZ-TIF2 induced AML. **A)** Hierarchical clustering and heat-map of the **49 shared activated probes** in MLL-ENL and MOZ-TIF2. The 49 probes were defined as outlined in Figure S3 in Supporting Information S1 and are derived from intersection of probes in the orange boxes of Figure S3D in Supporting Information S1. **B)** Hierarchical clustering and heat-map of the **111 shared repressed probes** in MLL-ENL and MOZ-TIF2. The 111 probes are derived from intersection of probes in the green boxes of Figure S3E in Supporting Information S1. **C)** Hierarchical clustering and heat-map of the **45 shared repressed probes** localised in the area indicated by white arrowheads in GEDI maps from the "leukaemic" cluster (Figure 2B). Note that in all three sets of shared probes, the samples always segregate into the previously mentioned 'non-leukaemic' and 'leukaemic' cluster, with MT-I sample-sets assigned to the 'non-leukaemic' and ME-I to the 'leukaemic' cluster. A list of the genes from each set can be found in Table S2 in Supporting Information S1.

Bonadies et al, Suppl. Figure 5

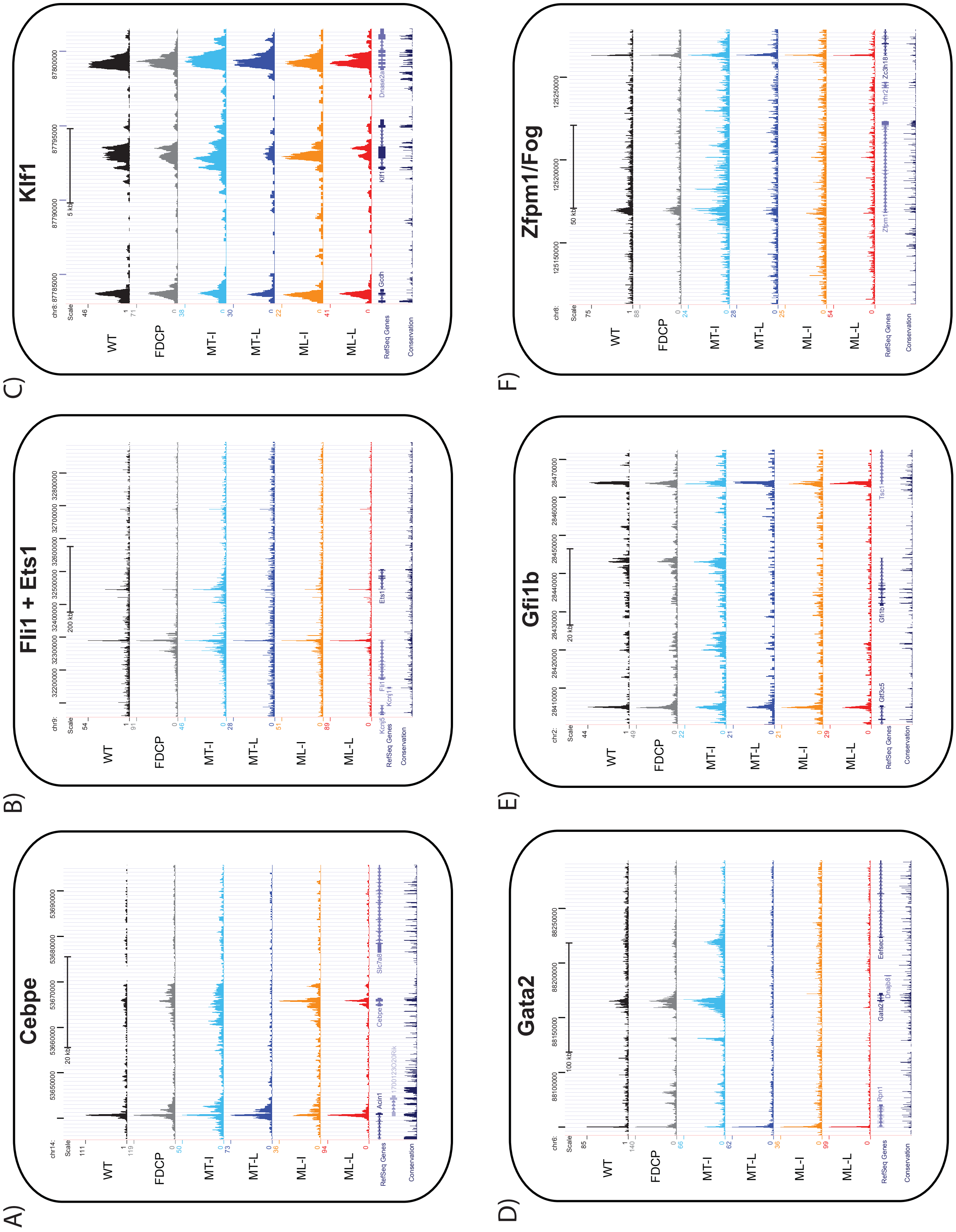

Supporting Figure S5 Histone acetylation ChIP-Seq traces of HSPC-TFs from the repression cluster depicted in Figure 3A. Representative ChIP-Seq traces for each sample condition are shown for HSPCTFs repressed in MOZ-TIF2-L only (Cebpe, Fli1) or repressed in both MLL-ENL and MOZTIF2 at leukaemic transition (Klf1, Gata2, Gfi1b, Zfpm1/Fog), Note that Ets1, showing a more variable pattern, is displayed together with Fli1, due to its close proximity in the murine genome.

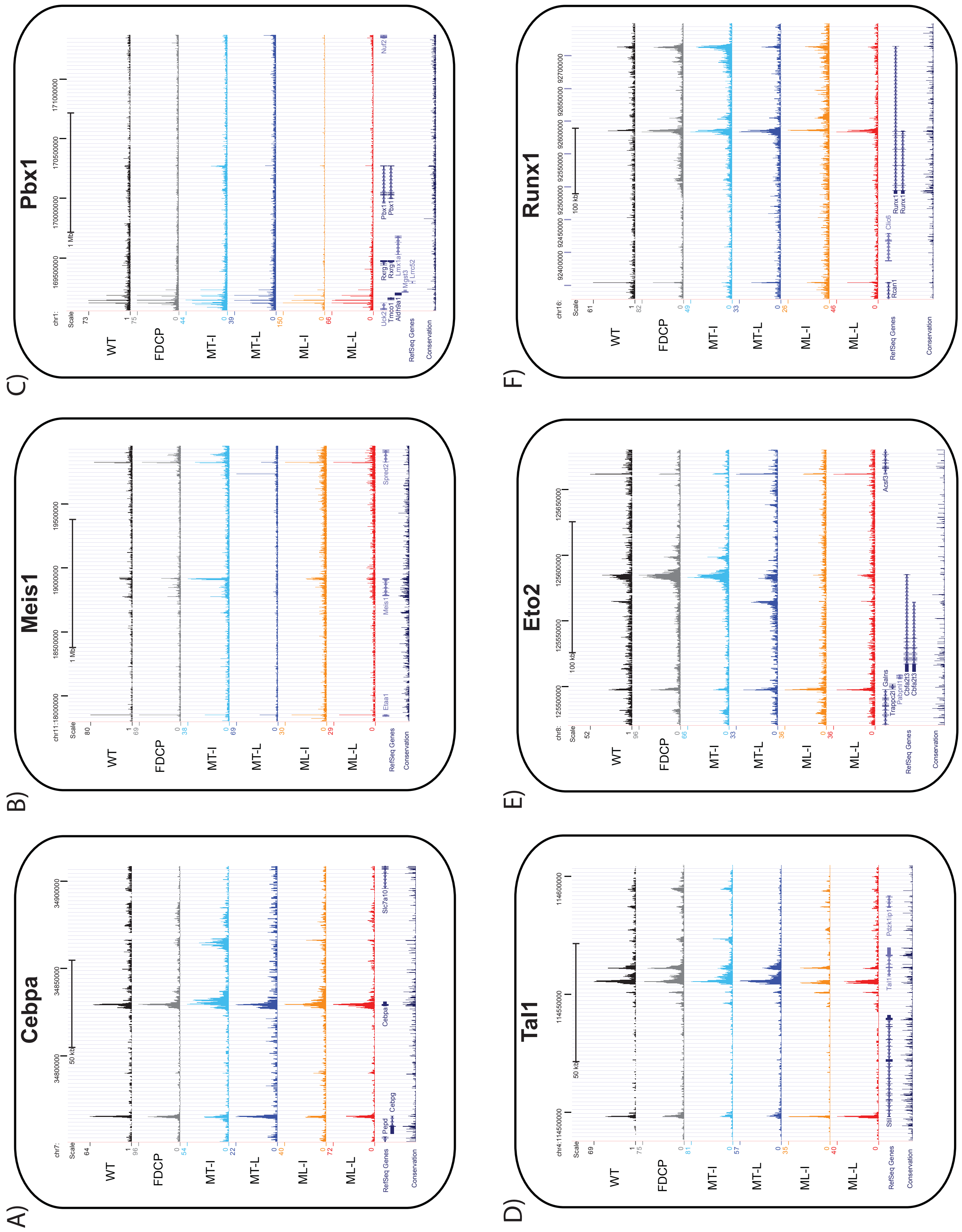

Supporting Figure S6 Histone acetylation ChIP-Seq traces of HSPC-TFs from the repression cluster depicted in Figure 3A. Representative ChIP-Seq traces for each sample condition are shown for HSPC7 TFs with variable patterns (Cebpa, Meis1, Pbx1) or repressed in MLL-ENL only (Tal1, Eto2, Runx1). Note that Ets1 (with variable pattern) is displayed with Fli1 in Figure S5 in Supporting Information S1.

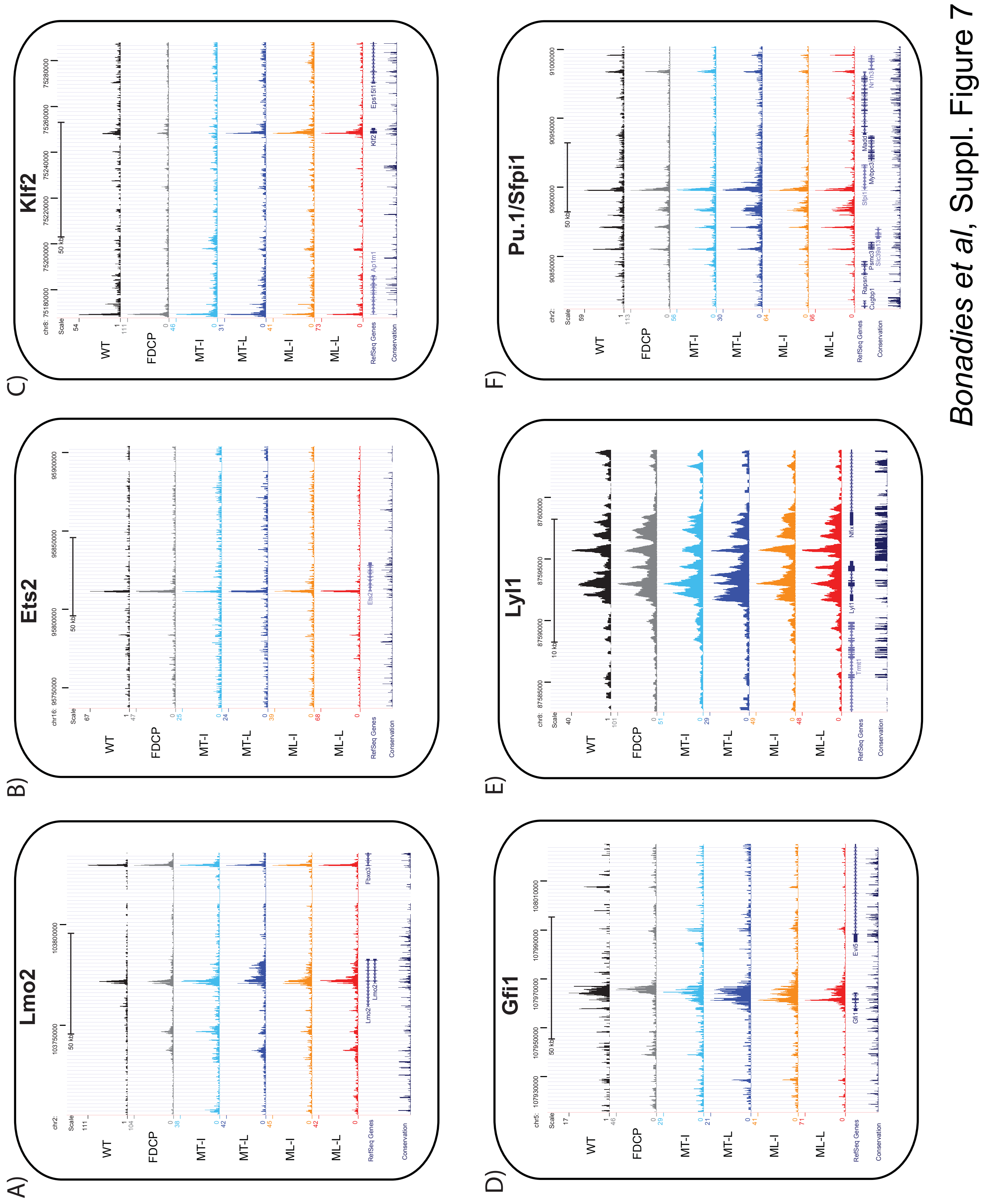

Supporting Figure S7 Histone acetylation ChIP-Seq traces of HSPC-TFs from the activation cluster depicted in Figure 3A. Representative ChIP-Seq traces for each sample condition are shown for HSPCTFs Lmo2, Ets2, Klf2, Gfi1, Lyl1 and Pu1/Sfpi1.

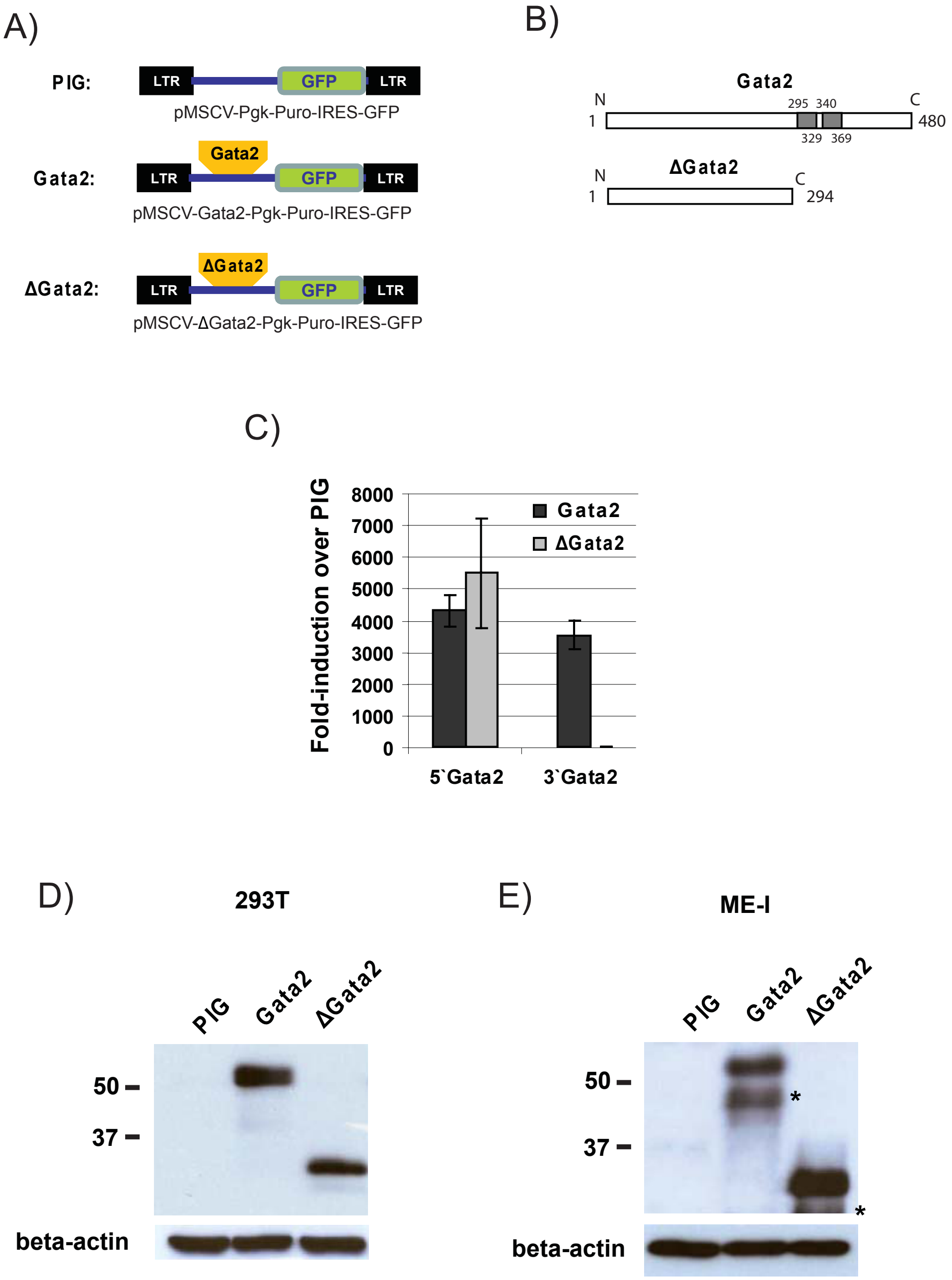

Supporting Figure S8 Validation experiments of retroviral Gata2 constructs used for competitive proliferation assays. **A)** GFP vector constructs used for retroviral over-expression: cDNA of full length Gata2 and _Gata2 were cloned with *BglII/XhoI* into *pMSCV-Pgk-Puro-IRES-GFP* (PIG), generating *pMSCV-Gata2-Pgk-Puro-IRES-GFP* (Gata2) and *pMSCV-_Gata2-Pgk-Puro- IRES-GFP* (_Gata2). **B)** Schema of Gata2 and _Gata2 proteins. Full length Gata2 contains 480 amino-acids, with two zinc-finger domains at position 295–329 and 340–369, respectively (*InterProSignature*: IPR000679/PF00320). _Gata2 is a 294 amino-acid long, N-terminal truncated protein, lacking the DNA-binding zinc-finger domains on the C-terminal region. **C)** QRT-PCR of ME-I cells sorted for GFP-positivity 36 hours after re-transduction with PIG, Gata2 and _Gata2 GFP-expression vectors. 5′and 3′ specific primers for Gata2 (5′Gata2 and 3′Gata2 with sequences available on request) were used to identify full length and deleted transcripts. X-axis depicts fold induction over PIG after normalization to Hprt1 and bars represent SD of two independent experiments. **D)** Western blot of transient transfected HET293T cells with PIG, Gata2 and _Gata2 GFP-expression vectors, showing the presence of the correct full length and deleted Gata2 proteins. **E)** Western blot of Gata2 of retroviral retransduced MLL-ENL-I cells with PIG, Gata2 and _Gata2 GFP-expression vectors, sorted for GFP-positivity 36 hours after transduction and showing the presence of the correct full length and truncated Gata2 proteins in re-transduced ME-I. * indicate potential degradation products of Gata2 and _Gata2.

Bonadies et al, Suppl. Figure 8

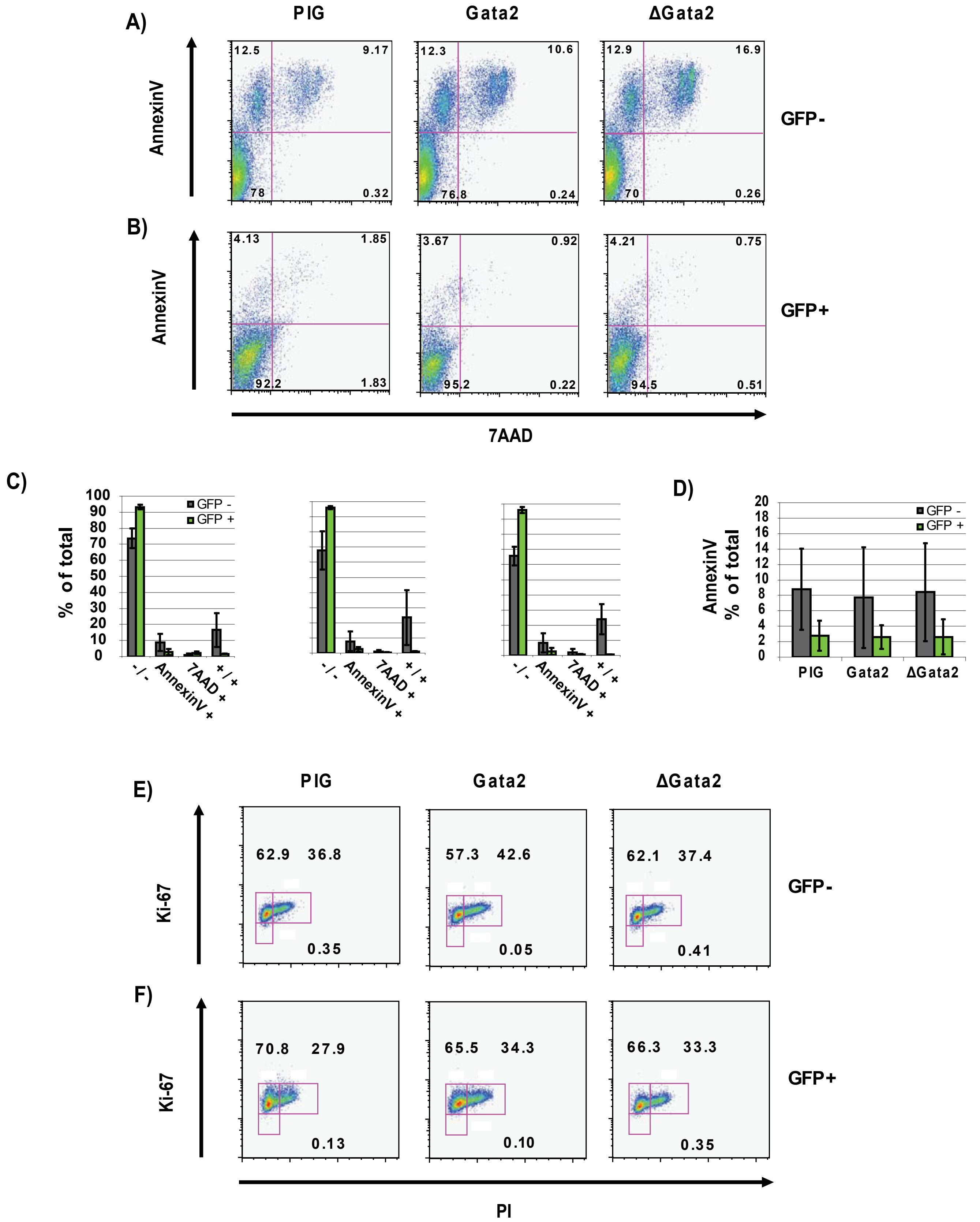

Supporting Figure S9 AnnexinV and Ki-67 staining of ME-I cells transduced with PIG, Gata2 and _Gata2 GFP expression vectors. **A)** AnnexinV staining 36 hours after infection for GFP-positive and GFP negative cells. Samples were run in parallel, without previous FACS-sorting. AnnexinV (Pacific Blue) and 7AAD fluorescence gated on GFP-negative cell-fraction. **B)** AnnexinV and 7AAD fluorescence gated on GFP-positive cell-fraction. **C)** Bar-charts summarizing the % of total number of GFP-positive and GFP-negative of gated cells for double negative (-/-), AnnexinV single positive (AnnexinV +), 7AAD single positive (7AAD +) and double positive (+/+) quadrants. Bars denote SD from two independent experiments. **D)** Different representation of C) combining the AnnexinV values for PIG, Gata2 and _Gata2 in one bar chart. **E)** Ki67 staining of ME-I cells re-transduced with PIG, Gata2 and _Gata2 GFP expression vectors. FACS analysis was performed 36 hours after infection and after sorting for GFP-positive and GFP-negative cells. Ki-67 (FITC) and Propidium Iodide (PE) fluorescence for GFP-negative cells. **F)** Ki-67 (FITC) and Propidium Iodide (PE) fluorescence for GFP positive cells.

Bonadies et al, Suppl. Figure 9

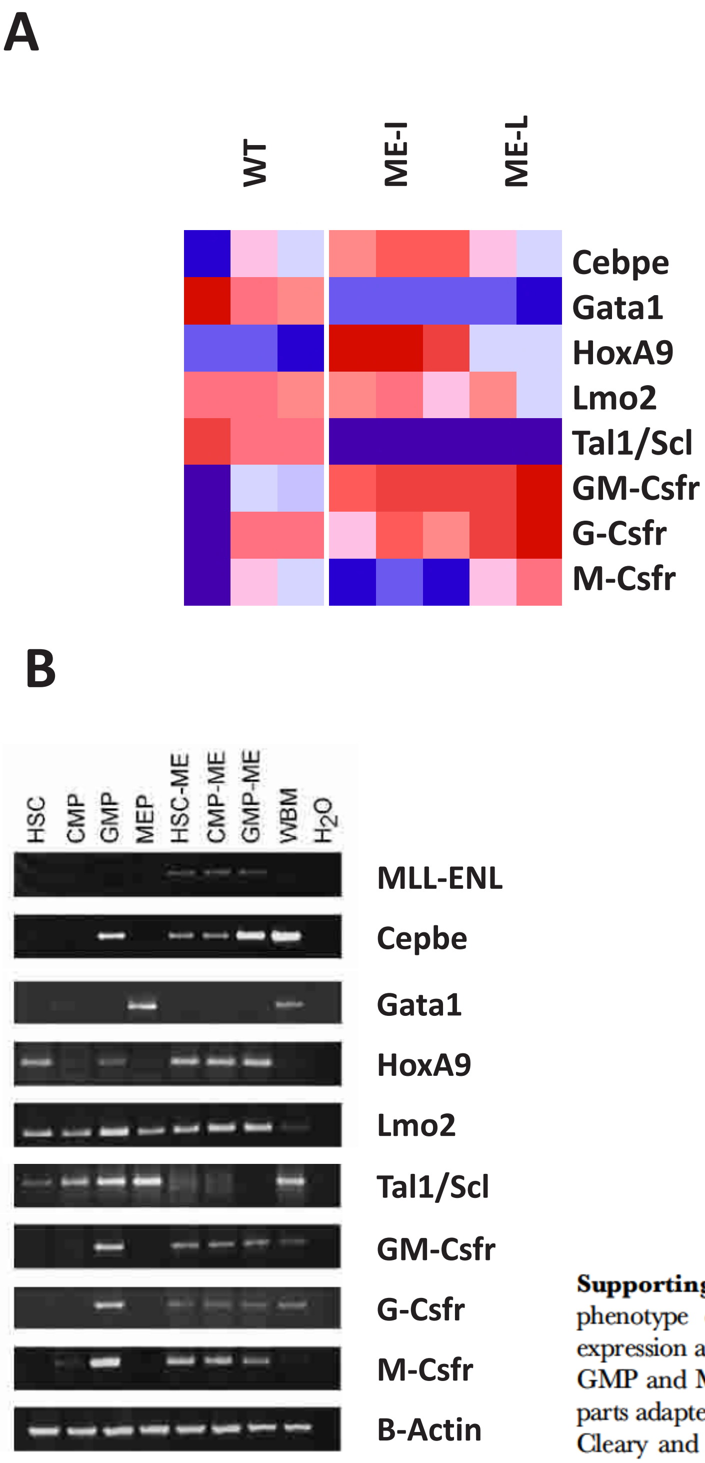

(adapted from A. Cozzio et al;
Genes Dev. 2003 17(24):3029-35)

Supporting Figure S10 **A)** Heatmap of selected HSPC TF and phenotype defining genes in WT, ME-I and ME-L. **B)** Gene expression analysis comparing HSC and myeloid progenitors (CMP, GMP and MEP) to heir MLL-ENL transduced leukaemic counterparts adapted from Cozzio et al (Cozzio, Passegue, Ayton, Karsunky, Cleary and Weissman 2003). The WT sample (Lin/c-kit+) used in our progression model contains a mixture of HSC, CMP, GMP cells. The expression profiles for HSPC-TF, such as *Cebpe*, *Gata1*, *Hoxa9*, *Tal1/Scl* and *Lmo2*, and the phenotypic markers, such as *GM-Csrf*, *G-Csfr* and *M-Csrf*, confirm the similarity of our transduced cells and the validity of our WT control.

Bonadies et al, Suppl. Figure 10

A) **Output from oPOSSUM**

FAQ | Help | About | News | Contact
oPOSSUM
Human SSA
Human CSA
Worm SSA
Yeast SSA
oPOSSUM Main | Home | Custom Analysis | Download API | Download DB

Human Single Site Analysis

Version 2.0

Analysis Results

Selected Parameters

Conservation level:	Top 10% of conserved regions (min. conservation 70%)
Matrix match score:	80%
Upstream sequence length:	5000
Downstream sequence length:	5000
Number of genes submitted:	75
Number of genes included:	55
Number of genes excluded:	20

Target Genes

Analyzed: Ptger3 Dapp1 Adrbk2 Rgs1 Aqp9 Nkg7 Usp10 Ptprs Pcyt1b Trfr2 Poli Padi2 Il1rl1 Rab38 Reep6 Nt5c3 Epx Casp9 Cbfa2t3h Stxbp1 Csrp3 Itgb7 Dtnb Mns1 Dyrk3 Msi2 Ugcg Ms4a2 Acss1 Angpt1 Prg3 Pank1 Tnik Gna14 Slc45a3 Stx3 Ndst2 Cpa3 Gfi1b Kcnk5 P2ry1 Muc13 Bahcc1 Tbc1d10c Fcer1a Prg2 Gp5 Gcnt1 F2r Ttc3 Gata2 Slc24a3 Dmwd AW146242 H2-Oa
Excluded: AI504432 AI875142 Cd55 Ear6 Epdr1 Hba-a1 Hbb-b1 Hdc Heatr5a Lat Mcpt8 Msi2h Plscr1 Prss34 Rec8 Siat7c Ssbp2 Zeb1 Zfp579 Zfp704

oPOSSUM Analysis

TF	TF Class	TF Supergroup	IC	Background gene hits	Background gene non-hits	Target gene hits	Target gene non-hits	Background TFBS hits	Background TFBS rate	Target TFBS hits	Target TFBS rate	Z-score	Fisher score
NF-kappaB	REL	vertebrate	13.345	5960	9190	30	25	11447	0.0050	62	0.0076	10.7	1.608e-02
MZF1_1-4	ZN-FINGER, C2H2	vertebrate	8.586	13090	2060	54	1	160814	0.0419	651	0.0479	8.632	3.155e-03
Cebpa	bZIP	vertebrate	9.187	8322	6828	40	15	23857	0.0124	106	0.0156	8.196	5.239e-03
HNF4A	NUCLEAR RECEPTOR	vertebrate	9.617	5541	9609	25	30	9566	0.0054	46	0.0073	7.543	1.113e-01
IRF2	TRP-CLUSTER	vertebrate	21.134	643	14507	5	50	709	0.0006	5	0.0011	6.607	8.422e-02
Myb	TRP-CLUSTER	vertebrate	9.883	10043	5107	46	9	36934	0.0128	157	0.0154	6.56	3.428e-03
REL	REL	vertebrate	10.515	7798	7352	33	22	18677	0.0081	82	0.0101	6.214	1.295e-01
Ar	NUCLEAR RECEPTOR	vertebrate	15.703	585	14565	3	52	619	0.0006	4	0.0011	5.671	3.583e-01
NFKB1	REL	vertebrate	15.627	2832	12318	17	38	4140	0.0020	21	0.0028	5.479	2.036e-02
STAT1	Stat	vertebrate	18.431	2165	12985	9	46	2693	0.0016	14	0.0024	5.39	3.866e-01

Download as a tab delimited text file (results will be kept on the server for 3 days after analysis)

Back to top

Consult the help file for an explanation of the column headings. To perform another analysis, please use the **Back** button on your browser or click the **Main** or **Custom Analysis** link at the top of the page to return to the **Select Analysis Parameters** form.

B)

Results from WebMOTIFS job aml (de novo MD) Page 1 of 1

Motif discovery was run on these sequences.

You requested motif discovery with AlignACE.
AlignACE discovered 341 motifs: Raw Program Output Motifs in TAMO text format

You requested motif discovery with MDscan.
MDscan discovered 25 motifs: Raw Program Output Motifs in TAMO text format

You requested motif discovery with Weeder.
Weeder discovered 4 motifs: Raw Program Output Motifs in TAMO text format

You requested motif discovery with MEME.
MEME discovered 6 motifs: Raw Program Output Motifs in TAMO text format

376 of these motifs were discarded as insignificant

No significant motifs found

Please note that this page will remain up for the next 30 days and then will be removed from out server. You can download the entire webpage (including all logos and intermediate output):wholepage.tar.gz.

Supporting Figure S11 Inference of transcriptional regulators from gene expression data alone using the shared repressed gene set from Table S2 in Supporting Information S1. A) The oPOSSUM tool identified 10 candidate overrepresented motifs but none correlated with differentially expressed HSPC TFs. B) The WebMOTIFS tool failed to identify any significant motifs.

Bonadies et al, Suppl. Figure 11

Supporting Table S1: Summary of differentially expressed probes.

	Not intersected			Intersected		
**Pair-wise comparisons**	**Total (% of all)**	**Up (% of total)**	**Down (% of total)**	**Total (% of all)**	**Up (% of total)**	**Down (% of total)**
A) MLL-ENL						
**1a. I v. WT**	**2073 (10%)**	980 (47.3%) *	1093 (52.7%) *	**724 (3.5%)**	342 (47.2%)	382 (52.8%)
**1b. I v. FDCP-mix**	**1953 (9.4%)**	780 (39.9%) ***	1173 (60.1%) ***			
**2. L v. I**	**186 (0.9%)**	107 (57.5%) *	79 (42.5%) *			
B) MOZ-TIF2						
**1a. I v. WT**	**1776 (8.6%)**	1019 (57.4%) ***	757 (42.6%) ***	**479 (2.3%)**	338 (70.6%)	141 (29.4%)
**1b. I v. FDCP-mix**	**1346 (6.5%)**	674 (50.1%) ns	672 (49.9%) ns			
**2. L v. I**	**2441 (11.8%)**	1109 (45.4%) ***	1332 (54.6%) ***			

Supporting Table S1 Summary of differentially expressed probes for six representative pair-wise comparisons using the MLL-ENL and MOZ-TIF2 mouse models. Numbers of differentially expressed probes at "initiation" (I) are shown for the two baseline samples WT (1a.) and FDCP-mix (1b.) and at "progression'' to frank leukaemia (L) (2.). The 'Not intersected' columns show total numbers in each of the six comparisons, as well as the corresponding numbers for up- and downregulated probes, respectively. Chi-Square Test was applied to test for significant changes between the up- and down-regulated probes: ns non significant, * <0.01, ** <0.001, *** <0.0001. Differentially expressed probes in WT and FDCP-mix were intersected to correct for *in-vitro* culturing artefacts and number of total, up- or down-regulated probes are shown in the 'Intersected' columns.

Supporting Table S 2: Differentially expressed genes shared at leukaemic transition between MLL-ENL and MOZ-TIF2.

Shared activated (n=40)	Shared repressed (n=88)		Shared repressed (GEDI) (n=23)
Aldh3b1	Acss1	Nt5c3	Aqp9
App	Adrbk2	P2ry1	Cpa3
B3gnt8	AI504432	Padi2	Csrp3
Bcl2a1b	AI875142	Pank1	Dnajc6
Bcl2a1c	Angpt1	Pcyt1b	Ear2
Bcl2a1d	Aqp9	Plscr1	Ear3
Camk1	AW146242	Poli	Ear6
Cask	Bahcc1	Prg2	F2r
Cdc14b	Casp9	Prg3	Fcer1a
Clec4d	Cbfa2t3h	Prss34	Gata2
Csf2ra	Cd55	Ptger3	Gfi1b
Ctbp2	Cpa3	Ptprs	Hba-a1
Cyp27a1	Csrp3	*Rab38*	Hbb-b1
Cysltr1	Dapp1	Rec8	Hdc
Gdpd3	Dmwd	Reep6	Mcpt8
Gnaq	Dtnb	Rgs1	Nkg7
Hexa	Dyrk3	Siat7c	P2ry1
Inppl1	Ear6	Slc24a3	Prss34
Lgals3	Epdr1	Slc45a3	LOC100044439
Lpl	Epx	Ssbp2	LOC100047651 (Zfpm1/Fog)
Mefv	F2r	Stx3	4930519L02Rik
Mlf1	Fcer1a	Stxbp1	5830405N20RIK
Mtus1	Gata2	Tbc1d10c	9830002I17RIK (Spns3)
Nt5e	Gcnt1	Tnik	
Oas1g	Gfi1b	Trfr2	
Pilra	Gna14	Ttc3	
Rapgef5	Gp5	Ugcg	
Retsat	H2-Oa	Usp10	
Rrbp1	Hba-a1	Zeb1	
Sh3tc1	Hbb-b1	Zfp579	
Snx24	Hdc	Zfp704	
Soat1	Heatr5a	LOC100047651 (Zfpm1/Fog)	
Tcfec	Il1rl1	1500005A01Rik (Fam158A)	
Tcirg1	Itgb7	1700123O20Rik)	
Tlr6	Kcnk5	2010001J22Rik (Odf3b)	
Unc93b1	Lat	2210410E06Rik (C1galt1)	
E430033B07Rik	Mcpt8	2610305D13Rik	
3300005D01Rik	Mns1	3830612M24	
6230401I02Rik (Gnaq)	Ms4a2	5330403D14Rik	
9530064J02 (Mical2)	Msi2	5430426F23Rik (Pank1)	
	Msi2h	5830405N20Rik	
	Muc13	9330175B01Rik (Slc45A4)	
	Ndst2	9630015D15Rik (Tmem64)	
	Nkg7	9830002I17Rik (Spns3)	

Supporting Table S2 Differentially expressed genes shared at leukaemic transition between MLL-ENL and MOZ-TIF2. List of genes products from shared activated, shared repressed and shared repressed GEDI probes at leukaemic transition of MLL-ENL and MOZ-TIF transduced cells. Underlined genes are found in both of the shared repressed gene-sets.
